# TCR Affinity Associated with Functional Differences between Dominant and Subdominant SIV Epitope-Specific CD8^+^ T Cells in Mamu-A*01 ^+^ Rhesus Monkeys

**DOI:** 10.1371/journal.ppat.1004069

**Published:** 2014-04-17

**Authors:** Christa E. Osuna, Ana Maria Gonzalez, Hsun-Hsien Chang, Amy Shi Hung, Elizabeth Ehlinger, Kara Anasti, S. Munir Alam, Norman L. Letvin

**Affiliations:** 1 Center for Virology and Vaccine Research, Beth Israel Deaconess Medical Center, Harvard Medical School, Boston, Massachusetts, United States of America; 2 Department of Environmental Health, Harvard School of Public Health, Boston, Massachusetts, United States of America; 3 Children's Hospital Informatics Program, Harvard-MIT Division of Health Sciences and Technology, Harvard Medical School, Boston, Massachusetts, United States of America; 4 Duke Human Vaccine Institute, Duke University School of Medicine, Durham, North Carolina, United States of America; 5 Department of Pathology, Duke University of Medicine, Durham, North Carolina, United States of America; National Institute of Health Vaccine Research Center, United States of America

## Abstract

Many of the factors that contribute to CD8^+^ T cell immunodominance hierarchies during viral infection are known. However, the functional differences that exist between dominant and subdominant epitope-specific CD8^+^ T cells remain poorly understood. In this study, we characterized the phenotypic and functional differences between dominant and subdominant simian immunodeficiency virus (SIV) epitope-specific CD8^+^ T cells restricted by the major histocompatibility complex (MHC) class I allele Mamu-A*01 during acute and chronic SIV infection. Whole genome expression analyses during acute infection revealed that dominant SIV epitope-specific CD8^+^ T cells had a gene expression profile consistent with greater maturity and higher cytotoxic potential than subdominant epitope-specific CD8^+^ T cells. Flow-cytometric measurements of protein expression and anti-viral functionality during chronic infection confirmed these phenotypic and functional differences. Expression analyses of exhaustion-associated genes indicated that LAG-3 and CTLA-4 were more highly expressed in the dominant epitope-specific cells during acute SIV infection. Interestingly, only LAG-3 expression remained high during chronic infection in dominant epitope-specific cells. We also explored the binding interaction between peptide:MHC (pMHC) complexes and their cognate TCRs to determine their role in the establishment of immunodominance hierarchies. We found that epitope dominance was associated with higher TCR:pMHC affinity. These studies demonstrate that significant functional differences exist between dominant and subdominant epitope-specific CD8^+^ T cells within MHC-restricted immunodominance hierarchies and suggest that TCR:pMHC affinity may play an important role in determining the frequency and functionality of these cell populations. These findings advance our understanding of the regulation of T cell immunodominance and will aid HIV vaccine design.

## Author Summary

MHC-restricted CD8^+^ T cell populations that bind viral proteins are often present at different frequencies. It is thought that those virus-specific CD8^+^ T cells that are present at the highest frequency are predominantly responsible for eliciting control of viral infections. While the number of virus-specific CD8^+^ T cells is undoubtedly important, the functionality of these cells may also play an anti-viral role. It is not known if high-frequency virus-specific CD8^+^ T cells are more functionally effective against viral infection than those present at low frequencies. In this study, we characterized the functional differences between the SIV-specific cells present at high versus low frequencies in rhesus monkeys infected with simian immunodeficiency virus (SIV). We found that the high- and low-frequency SIV-specific cells had different functional capacities during acute and chronic SIV infection. We also found that the affinity with which a cell interacts with viral proteins may contribute to these functional differences. These findings further our understanding of anti-viral immune responses and may help to inform HIV vaccine development.

## Introduction

Virus-specific CD8^+^ T cells contribute to the control of HIV and SIV replication [1], [2] and are therefore an important component of a protective immunity. In infected or vaccinated individuals, the frequencies of different viral epitope-specific CD8^+^ T cells vary considerably [3], [4]. Epitopes restricted by the same MHC Class I allele can be ranked in an immunodominance hierarchy based on the relative frequencies of their respective epitope-specific CD8^+^ T cells [5]. The determinants of immunodominance hierarchies have been explored in the past in efforts to enhance the magnitude of particular epitope-specific CD8^+^ T cell responses through vaccination [6], [7]. However, such investigations have primarily focused on determining the mechanisms underlying the establishment of immunodominance [8]–[12]; less is known about the resulting functional differences between dominant and subdominant epitope-specific CD8^+^ T cells.

CD8^+^ T cell polyfunctionality is often associated with superior viral control [13]. Numerous studies have reported that individuals with superior control of HIV replication and delayed disease progression have higher frequencies of polyfunctional CD8^+^ T cells and therefore such cells are thought to be an important component of a protective immune response during HIV infection [14]–[17]. Therefore, HIV vaccine development is particularly focused on the identification of vaccine strategies that will generate highly polyfunctional, and therefore protective, responses [18]. In order to do so, a better understanding of the factors that contribute to the polyfunctionality of a CD8^+^ T cell response is needed.

Few studies have evaluated the functional differences between dominant and subdominant epitope-specific CD8^+^ T cells. Studies in DNA-immunized mice showed that subdominant epitope-specific CD8^+^ T cells were less cytotoxic, but produced more cytokines, than dominant epitope-specific CD8^+^ T cells during acute infection [19]. Moreover, a cryptic epitope-specific CD8^+^ T cell population exhibited an altered maturation phenotype when compared to cells comprising the dominant response [20]. A study of the functionality of CD8^+^ T cells within the HLA-B27-restricted immunodominance hierarchy in HIV-infected individuals found higher-frequency epitope-specific CD8^+^ T cells were associated with superior *in vitro* viral suppression; although, subsequent functional studies could not identify a particular function associated with this suppressive capacity [21]. Additional knowledge of functional differences between dominant and subdominant epitope-specific CD8^+^ T cell responses is needed for enhancing CD8^+^ T cell epitope-specific responses by vaccination.

In the present studies, we performed longitudinal gene expression analysis in SIV-infected Mamu-A*01 ^+^ rhesus monkeys to assess potential functional differences between dominant and subdominant epitope-specific CD8^+^ T cells during acute infection. We demonstrate that dominant epitope-specific CD8^+^ T cells display gene expression patterns consistent with a more mature phenotype and harbor greater cytotoxic potential than subdominant epitope-specific CD8^+^ T cells during acute infection. Protein expression analyses of CD8^+^ T cells sampled during chronic infection confirmed the presence of these functional differences between dominant and subdominant epitope-specific CD8^+^ T cells. Finally, dominant and subdominant epitope TCR:pMHC affinities were correlated with relative immunodominance. These findings advance our understanding of the basis underlying immunodominance and may better inform T cell-based HIV vaccine design.

## Results

### p11C- and p54AS-specific CD8^+^ T cell frequencies and viral loads during acute SIVmac251 infection

We infected six Mamu-A*01 ^+^ rhesus monkeys with SIVmac251. The Mamu-A*01-restricted dominant SIV Gag p11C- and subdominant SIV Env p54AS-specific CD8^+^ T cells were quantified and sorted by flow cytometry on days 14, 21, 28, 35, 42, 56, and 70 post-inoculation. The Mamu-A*01-restricted CD8^+^ T cell response is one of the best characterized CD8^+^ T cell responses in SIV-infected rhesus monkeys [22], [23]. The p11C epitope (Gag CM9) has been used widely as a model dominant epitope in studies of SIV-specific immunity [24]–[26]. p54AS (Env TL9) is a well-described subdominant epitope that, from our experience, was the most likely to be elicited in all Mamu-A*01 ^+^ rhesus monkeys at a frequency that was high enough such that we could collect sufficient numbers of cells to facilitate our gene expression measurements.

**Figure 1:**
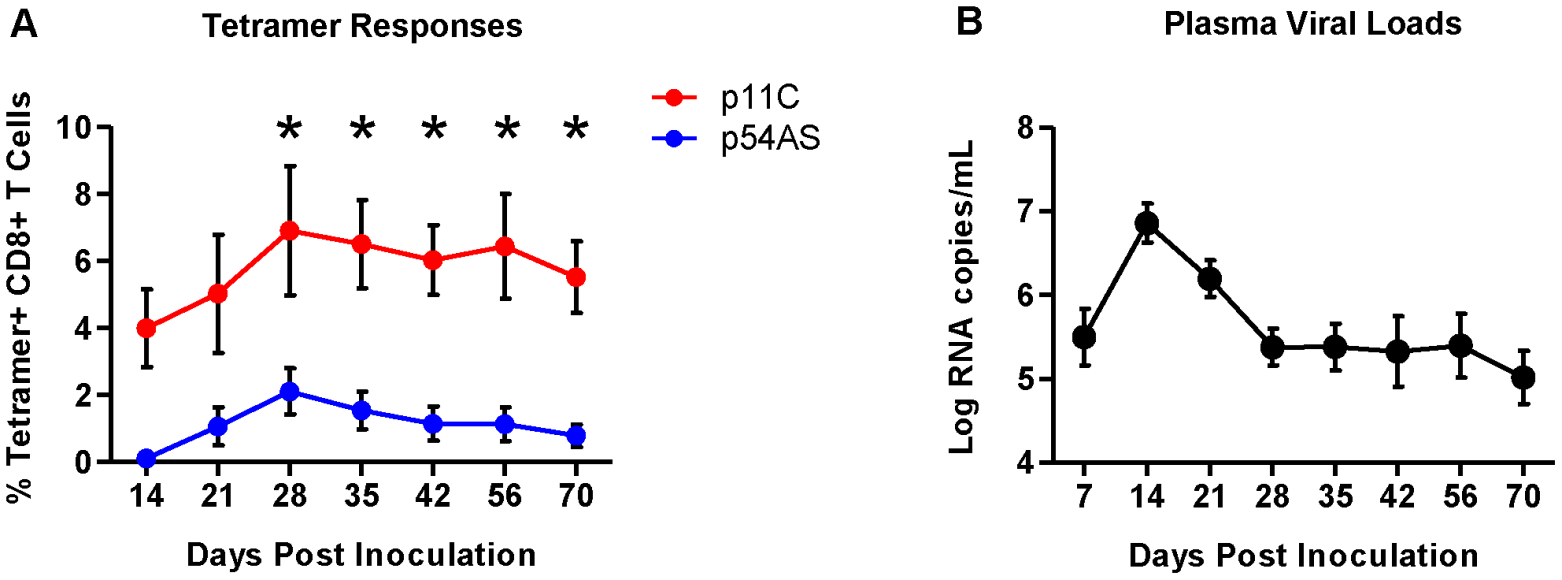
Frequencies of p11C- and p54AS-specific CD8^+^ T cells and plasma viral loads during primary infection. A) Mean frequencies of the p11C- and p54AS-specific CD8^+^ T cells in peripheral blood. * significant at *p*≤0.05. B) Plasma SIV RNA levels in the peripheral blood.

The differences in frequency between the p11C- and p54AS-specific CD8^+^ T cells were apparent upon detection in the peripheral blood (day 14) and were maintained throughout the duration of infection (Figure 1A and Figure S1). The plasma viral loads peaked at approximately 7 logs of viral RNA copies/mL by day 14 and reached setpoint of approximately 5.5 logs of viral RNA/mL by day 28 (Figure 1B).

We measured RNA expression on the sorted epitope-specific cell populations at various timepoints post infection. We also sorted total naïve CD8^+^ T cells (CD95^−^CD28^+^) before challenge (day 0) and measured RNA expression in these cells to establish a baseline expression level for each transcript. We generated expression data from both epitope-specific cell populations from all six monkeys only on days 14, 21, 56 and 70. We obtained expression data for both epitope-specific populations in only some of the monkeys on the three other time points: days 35 and 42 (4 of 6 monkeys) and day 28 (5 of 6 monkeys). Therefore, sometimes we observed trends suggesting differential expression (Figure S2) that did not achieve statistical significance possibly due to reduced power.

To characterize the functional differences between the dominant p11C- and subdominant p54AS-specific cells based on their RNA expression profiles, we focused expression analysis on genes known to be associated with specific CD8^+^ T cell functions. These genes were grouped into the following categories: maturation, cytotoxicity, cell cycle and apoptosis, and cytokines and chemokines (Table 1). Genes were considered differentially expressed if they satisfied both of the following criteria: 1) expression was significantly different between the two epitope-specific cells on at least one timepoint and 2) the median fold difference of expression at this timepoint (p11C/p54AS) was at least ±1.5. Technical replicates were not conducted; gene expression was measured once per monkey per timepoint.

**Table 1:** Genes compared between dominant and subdominant epitope-specific CD8^+^ T cells.

Maturation	Cytotoxicity	Cytokines and Chemokines	Cell Cycle and Apoptosis					
B3GAT1	AP3B1	CCL3	AIFM1	BRCA2	CDKN1A	HDAC2	NAIP	SMAD2
BCL6	C1ORF9	TNFSF11	AIFM2	BTRC	CDKN1B	HDAC3	NCAPD2	SMAD3
BMI1	CSPG5	CCL7	AKT1	CABIN1	CDKN1C	HRAS	NCAPG	SMAD4
CCR7	CTSC	CCL4	ANAPC1	CASP1	CDKN2A	HRK	NCAPH	SMAD5
CD27	EBAG9	CCL5	ANAPC10	CASP10	CDKN2B	HTRA2	NFKB1	SMAD6
CD28	GNLY	TGFB1	ANAPC11	CASP12	CDKN2C	HUS1	NFKB2	SMAD7
CXCR3	GZMA	IL12A	ANAPC13	CASP2	CDKN2D	ID1	NOL3	SMAD9
EOMES	GZMB	MIF	ANAPC16	CASP3	CEBPA	ID2	NRAS	SMC2
GFI1	GZMH	IL22	ANAPC2	CASP6	CEBPB	ID3	NUSAP1	SMC4
ID2	GZMK	IL16	ANAPC4	CASP7	CENPF	ID4	PCNA	SNRPE
IL2RA	IQGAP1	IL26	ANAPC5	CASP8	CFLAR	IGF1	PEA-15	SOD2
IL7R	JAKMIP1	IL29	ANAPC7	CASP9	CHEK1	IGFBP3	PKMYT1	SP1
ITGAL	LAMP2	IL1A	APAF1	CCNA1	CHEK2	IRF1	PLK1	STK11
KLF2	LYST	IFNW1	APC	CCNA2	CKS1B	IRF2	PMAIP1	SUV39H1
KLRG1	M6PR	IL10	ARAF	CCNB1	CKS2	IRF3	PPARA	TACC3
MBD2	PRF1	SPP1	ARHGAP19	CCNB2	CPEB1	IRF4	PPKACA	TCL1A
PRDM1	RAB27A	TNFSF14	ATM	CCNC	CUL1	IRF8	PPKACB	TERT
SELL	SMPD1	IFNB1	ATR	CCND1	DAP	IRF9	PRKAR1A	TFDP1
SPN	SNAP23	XCL2	AURKB	CCND2	DAXX	JUN	PRKAR1B	TFDP2
TBX21	SNAP25	XCL1	BAD	CCND3	DIABLO	JUNB	PRKAR2A	THAP5
XBP1	SRGN	OSM	BAK1	CCNDBP1	DLG1	KAT2B	PRKAR2B	TNFR1
	STOML2	CX3CL1	BARD1	CCNE1	DLG3	KIT	PRKDC	TNFRSF10A
	STX11	CCL20	BAX	CCNE2	DLG4	KLF10	PRPS1	TNFRSF10B
	STXBP2	IL17D	BBC3	CCNF	E2F1	KLF4	RAC1	TNFRSF21
	SYTL1	IL17F	BCL2	CCNG2	E2F2	KLF5	RAD1	TNFRSF25
	SYTL2	IL17A	BCL2A1	CCNH	E2F3	KLF6	RAF1	TNFSF10
	TFF1	IFNG	BCL2L1	CDC16	E2F4	KRAS	RALA	TP53
	TRIP10	LTB	BCL2L10	CDC20	E2F5	MAP2K1	RALB	TP53AIP1
	UNC13D	LTA	BCL2L11	CDC23	E2F6	MAP2K2	RALGDS	TP73
	VAMP7	TNF	BCL2L14	CDC25A	E2F7	MAP3K5	RB1	TRADD
	VAMP8	FASLG	BCL2L2	CDC25B	ECT2	MAP3K7	RBL1	TRAF2
		CXCL11	BCL3	CDC25C	EGR1	MAPK1	RBL2	TRIM25
		CXCL10	BCL6	CDC27	EIF4EBP1	MAPK14	REL	TSC1
		IL18	BID	CDC2L2	ENDOG	MAPK7	RELA	TSC2
		CXCL5	BIK	CDC42	EP300	MAX	RELB	TSC22D1
		IRF4	BIN1	CDC42EP4	FADD	MCL1	RGL1	TTK
		CCL18	BIRC2	CDCA8	FAS	MDM2	RGL2	UBE2C
		IFNA1	BIRC3	CDK1	FASLG	MDM4	RHEB	UHRF1
		IL13	BIRC5	CDK10	FBX05	MEF2A	RHEBL1	WEE1
		CSF2	BIRC6	CDK11A	FLT3	MEF2B	RHOA	XIAP
		IL6	BIRC7	CDK11B	FOSL1	MEF2C	RING1	YWHAB
		CXCL9	BIRC8	CDK13	FOSL2	MEF2D	RPS6KB1	YWHAE
		IL4	BLK	CDK16	FOXO1	MGA	RPS6KB2	YWHAG
		IL5	BMF	CDK2	FOXO3	MKI67	RRAS	YWHAH
		IL2	BNIP3	CDK3	FOXO4	MNAT1	RRAS2	YWHAQ
		IL3	BNIP3L	CDK4	FZR1	MXD1	SCRIB	YWHAZ
		IL8	BOK	CDK5	GABPA	MXD3	SFN	YY1
			BRAF	CDK6	GADD45A	MYB	SKP2	ZMIZ1
			BRCA1	CDK7	HDAC1	MYC	SMAD1	

### Maturation-associated gene expression

**Figure 2:**
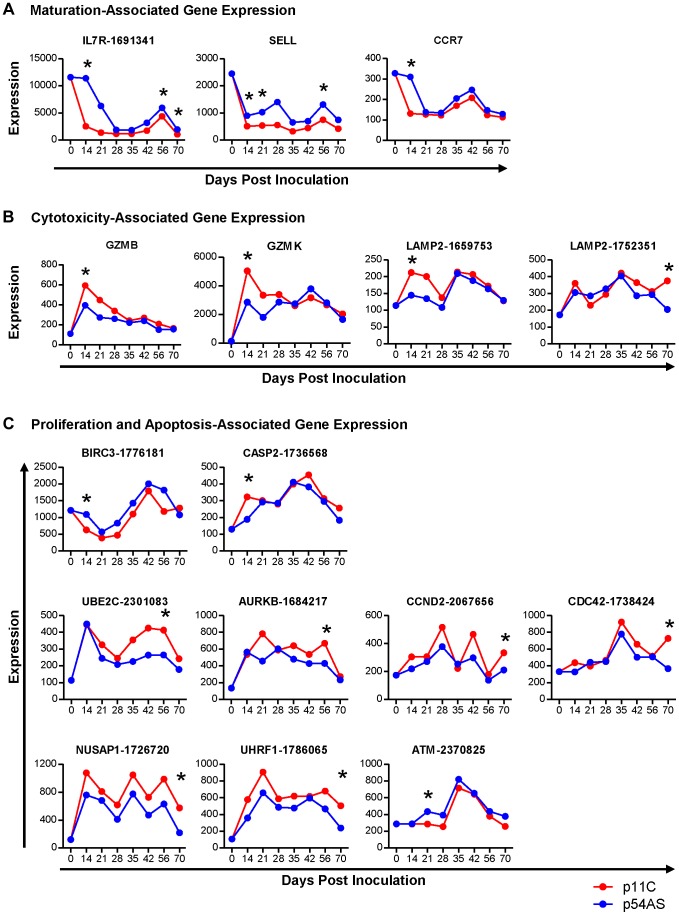
Genes differentially expressed between dominant p11C- and subdominant p54AS-specific CD8^+^ T cells. The median of the normalized expression values, measured in fluorescence units, of A) maturation, B) cytotoxicity, and C) proliferation and apoptosis genes that were found to be differentially expressed between the p11C- and p54AS-specific cells on at least one time point are shown for each timepoint evaluated during the first 70 days following SIVmac251 infection. The expression of these genes by total naïve CD8^+^ T cells, measured on day 0, is also shown. For those genes with more than one probe on the BeadChip, the numerical probe IDs are included in the gene name. * indicates that expression met the criteria for differential expression on that timepoint.

Of the genes involved in CD8^+^ T cell maturation, *IL7R* (CD127/IL-7 receptor, refseq accession # XM_937367.1), *SELL* (CD62L/L-selectin, NM_000655.3), and *CCR7* (NM_001838.2) met our criteria for differential expression following SIV infection (Figure 2A). While the trends of expression of these three genes between p11C- and the p54AS-specific cells were similar over time, all of these genes were found to be expressed at relatively higher levels in the subdominant p54AS-specific cells compared to the dominant p11C-specific cells. *IL7R* expression was higher in the p54AS-specific cells with significant fold differences of 1.8 on day 14, 1.5 on day 56, and 1.9 on day 70. *SELL* expression was also higher in the p54AS-specific cells with significant fold differences of 1.8 on day 14, 2.0 on day 21, and 1.7 on day 56. *CCR7* was more highly expressed in the p54AS-specific cells with a significant fold difference of 2.4 on day 14. While the expression of *IL7R* and *SELL* tended to be higher in the p54AS-specific cells for all timepoints examined, the expression for *CCR7* appeared only to be different on day 14 (Figure 2A and Figure S2A).

### Cytotoxicity-associated gene expression

Of the genes involved in cytotoxicity, *GZMB* (granzyme B, NM_004131.3), *GZMK* (granzyme K, NM_002104.2), and *LAMP2* (CD107b, NM_013995.1) met our criteria for differential expression following SIV infection (Figure 2B). *LAMP2* was measured on the BeadChip by three different probes, two of which measured differential expression (probes 1659753 and 1752351). Expression levels of each of the three cytotoxicity-associated genes were found to be increased in the dominant p11C-specific cells compared to the subdominant p54AS-specific cells. On day 14, the p11C-specific cells exhibited higher expression of both *GZMB* (significant fold difference of 1.5) and *GZMK* (significant fold difference of 1.9). Although the trends in differential expression of these two granzyme genes were still apparent on day 21 and 28, differences were subsequently lost (Fig. 2B and Figure S2B). The two *LAMP2* probes detected similar trends of expression of this gene. However, probe 1659753 detected significant differential expression on day 14 (1.5-fold) while probe 1752351 detected significant differential expression on day 70 (1.8-fold).

### Cell cycle and apoptosis-associated gene expression

Of the cell cycle and apoptosis-associated genes, nine met our criteria for differential expression following SIV infection (Figure 2C and Figure S2C). Two of these genes, *BIRC3* (AIP1/API2/c-IAP2, NM_001165.3) and *CASP2* (caspase-2, NM_032983.2), are involved in apoptosis. *BIRC3*, which encodes a protein with multiple anti-apoptotic functions [27], was differentially expressed on day 14, showing 1.8-fold higher expression in the p54AS-specific cells. *CASP2*, which encodes a pro-apoptotic protease [28], was differentially expressed on day 14, showing 1.7-fold higher expression in the p11C-specific cells.

The remaining seven differentially expressed genes are involved in proliferation: *UBE2C* (ubiquitin-conjugating enzyme E2C, NM_181800.1), *AURKB* (aurora B kinase, NM_004217.2), *CCND2* (cyclin D2, NM_001759.2), *CDC42* (cell division cycle 42, NM_001039802.1), *NUSAP1* (nucleolar and spindle associated protein 1, NM_018454.5), *UHRF1* (ubiquitin-like with PHD and ring finger domains 1, NM_001048201.1), and *ATM* (ataxia telangiectasia, NM_000051.3). *UBE2C* and *AURKB* encode pro-proliferative genes involved in the regulation of the anaphase-promoting complex [29], [30]; both were more highly expressed in the p11C-specific cells on day 56 with 1.6- and 1.5- fold differences, respectively. *CCND2*, *CDC42*, *NUSAP1*, and *UHRF1* also encode pro-proliferative genes [31]–[35]. All of these genes showed differential expression on day 70 and were more highly expressed in the p11C-specific cells: *CCND2* by 1.6-fold, *CDC42* by 1.9-fold, *NUSAP1* by 2.7-fold, and *UHRF1* by 2.5-fold. Finally, *ATM*, an anti-proliferative serine/threonine protein kinase [36], was differentially expressed on day 21 and was more highly expressed in the p54AS-specific cells with by 1.5-fold.

In summary, we detected differential expression of genes involved in CD8^+^ T cell maturation, cytotoxicity, and cell cycle and apoptosis suggesting that functional differences may exist between the dominant p11C-specific and the subdominant p54AS-specific cells. Decreased expression of the genes encoding CD127, CD62L, and CCR7 and the increased expression of genes encoding granzyme B, granzyme K, and CD107b (LAMP2) in the dominant p11C-specific cells compared to the subdominant p54AS-specific cells suggested that the p11C-specific cells were more mature and possessed greater cytotoxic potential than the p54AS-specific cells. Moreover, we observed that the p11C-specific cells had higher expression of pro-proliferative genes and an increased expression of a pro-apoptotic molecule.

### The dominant epitope-specific CD8^+^ T cell population was enriched with cells that exhibit a more mature phenotype

**Figure 3:**
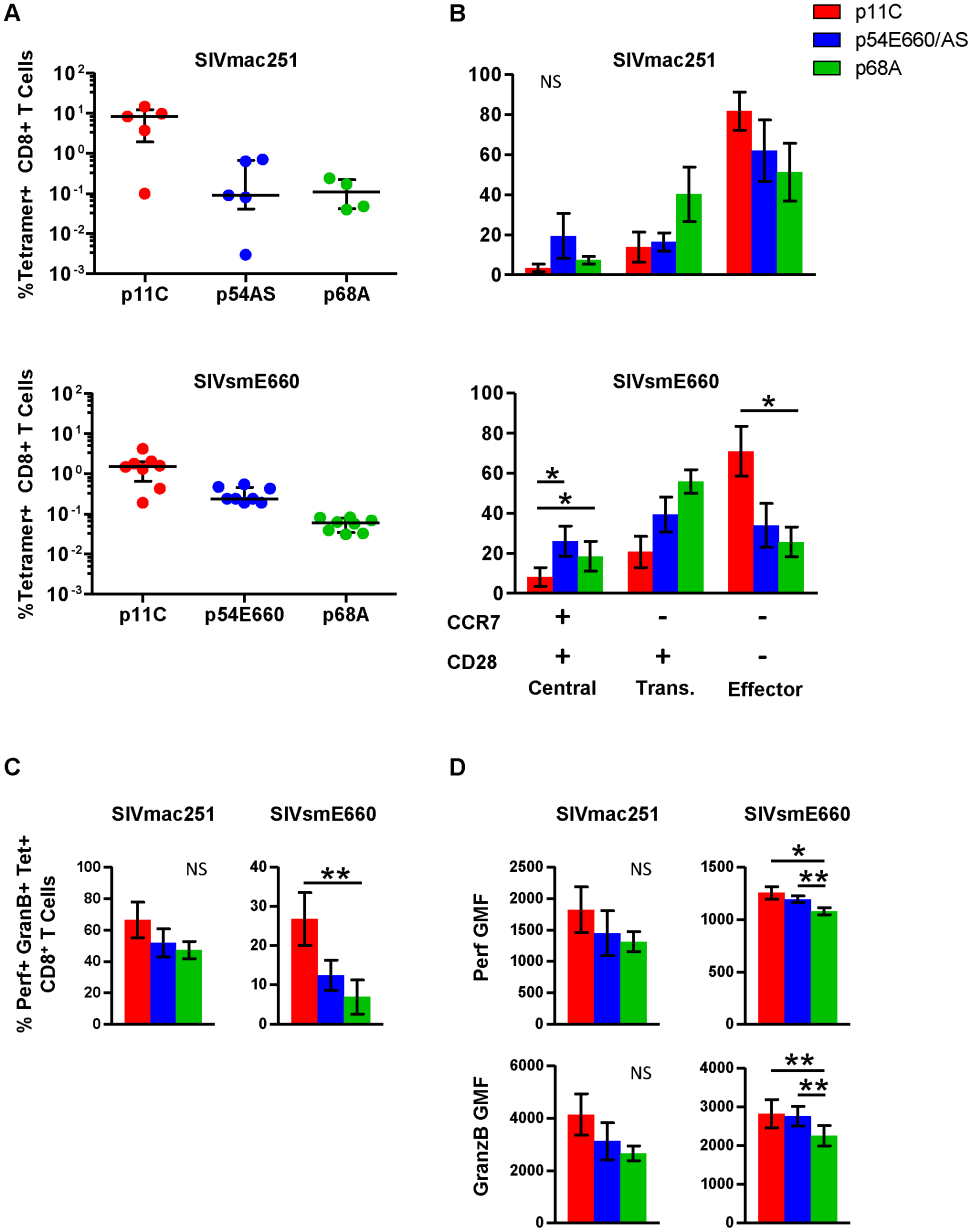
Phenotype and cytotoxic potential of dominant and subdominant epitope-specific cells during chronic SIVmac251 and SIVsmE660 infection. The frequency, cell surface phenotype, and *ex vivo* perforin and granzyme B content were evaluated for p11C-, p54AS/E660-, and p68A-specific cells, identified with Mamu-A*01 tetramers, from peripheral blood of chronically infected SIVmac251- and SIVsmE660- infected rhesus monkeys. A) Frequencies of the p11C-, p54AS/E660-, and p68A-specific cells from (top) SIVmac251- and (bottom) SIVsmE660- infected rhesus monkeys. Error bars indicate median ± interquartile range. SIVmac251 frequencies measured between weeks 37–50, except for 133-06 which died early and data presented are from week 18. SIVsmE660 frequencies are measured between weeks 19–22. B) Phenotyping of cells from SIVmac251- (top, n = 4) and SIVsmE660-infected monkeys (bottom, n = 8) based on cell surface expression of CCR7 and CD28. Positivity for CCR7 and CD28 is indicated by the + and − signs below the bar graph. Cells were categorized as central memory (CCR7^+^CD28^+^), transitional memory (CCR7^−^CD28^+^), or effector memory (CCR7^−^CD28^−^). All tetramer-positive cells were CD95^+^. Phenotyping was conducted between weeks 44–78 for SIVmac251 and 33–46 for SIVsmE660. C) Measurement of percent of *ex vivo* perforin^+^granzyme B^+^ cells from SIVmac251- (Left, n = 5) or SIVsmE660-infected monkeys (right, n = 8). D) Per-cell expression of perforin and granzyme B, measured by geometric mean of fluorescence (GMF) of perforin and granzyme B staining on cells from SIVmac251- (left, n = 5) and SIVsmE660-infected monkeys (right, n = 8). Perforin and granzyme B measurements were conducted between weeks 63–83 for SIVmac251 and 41–49 for SIVsmE660. In parts B–D, error bars represent mean ± SEM. * and **, significant at *p*≤0.05 and 0.01, respectively, after Bonferroni correction. NS, not significant.

Functional assays were performed on PBMCs isolated from chronically infected monkeys to confirm and extend the observations of differential gene expression patterns. In addition to the dominant p11C- and subdominant p54AS-specific cells, we studied an additional CD8^+^ T cell population of even lower frequency that recognizes the Mamu-A*01-restricted SIV Pol p68A (STPPLVRLV, Pol SV9_621_) subdominant epitope [23]. Additionally, we analyzed cells from monkeys infected with a different SIV isolate, SIVsmE660 [37]. The p54 epitope from the SIVsmE660 (TVPWPNETL) virus differs by two amino acids from the p54 epitope from SIVmac251 (TVPWPNASL) virus [22] and we refer to this epitope as p54E660. The immunodominance hierarchy of the p11C-, p54AS/E660-, and p68A-specific cells during chronic SIVsmE660 infection was similar to SIVmac251-infected monkeys (Figure 3A and Figure S3).

The maturation of tetramer-positive cell populations from SIVmac251- and SIVsmE660-infected monkeys was determined by measuring the surface expression of CCR7 and CD28. Cells were categorized as central memory (CCR7^+^CD28^+^), transitional memory (CCR7^−^CD28^+^), or fully differentiated effector memory (CCR7^−^CD28^−^) as previously described for rhesus monkeys [38]. All tetramer-positive cells were CD95^+^ (not shown). The dominant p11C-specific cells displayed a greater proportion of the more mature effector memory phenotype (CCR7^−^CD28^−^) compared to the subdominant p54AS-specific cells (Figure 3B and Figure S4). This is consistent with gene expression studies in SIVmac251-infected monkeys that expressed *CCR7*, *CD62L*, and *SELL* at lower levels in the p11C-specific cells, suggesting they were more mature than the p54AS-specific cells.

Additionally, we found that the more subdominant p68A-specific cells were phenotypically less mature compared to the subdominant p54AS/E660-specific cells (Figure 3B). Of the three epitope-specific cell populations, the p68A-specific population was the most enriched with cells of the less mature phenotypes and were the least enriched with cells of the mature effector memory phenotype. This trend was observed in both SIVmac251- and SIVsmE660-infected monkeys. While the distribution of phenotypes was similar between cells from SIVmac251- and SIVsmE660-infected monkeys, the sample size for SIVmac251 (n = 4) was much smaller than that of SIVsmE660 (n = 8) and was not sufficiently powered to detect significance.

To confirm that differences in gene expression of cytolytic molecules were similarly reflected in protein production, we measured the intracellular content of perforin and granzyme B within each tetramer-positive population. The dominant p11C-specific populations had a greater frequency of cells containing perforin and granzyme B than the subdominant p54AS/E660-specific population, which in turn had a greater frequency of perforin- and granzyme B-containing cells than the subdominant p68A-specific population (Figure 3C and Figure S5). The p11C-specific cells also contained more perforin and granzyme B per-cell (reflected by higher geometric mean fluorescence (GMF)) than the p54AS/E660-specific cells, which in turn contained more of these proteins than the p68A-specific cells (Figure 3D and Figure S5). These observations were consistent with previous findings that demonstrated not only that more mature cells are more likely to express perforin and granzymes [39], but also that more mature cells have more perforin and granzyme per cell than less mature cells [40].

### The dominant p11C-specific CD8^+^ T cells exhibited decreased antigen-specific expansion compared to subdominant epitope-specific CD8^+^ T cells

**Figure 4:**
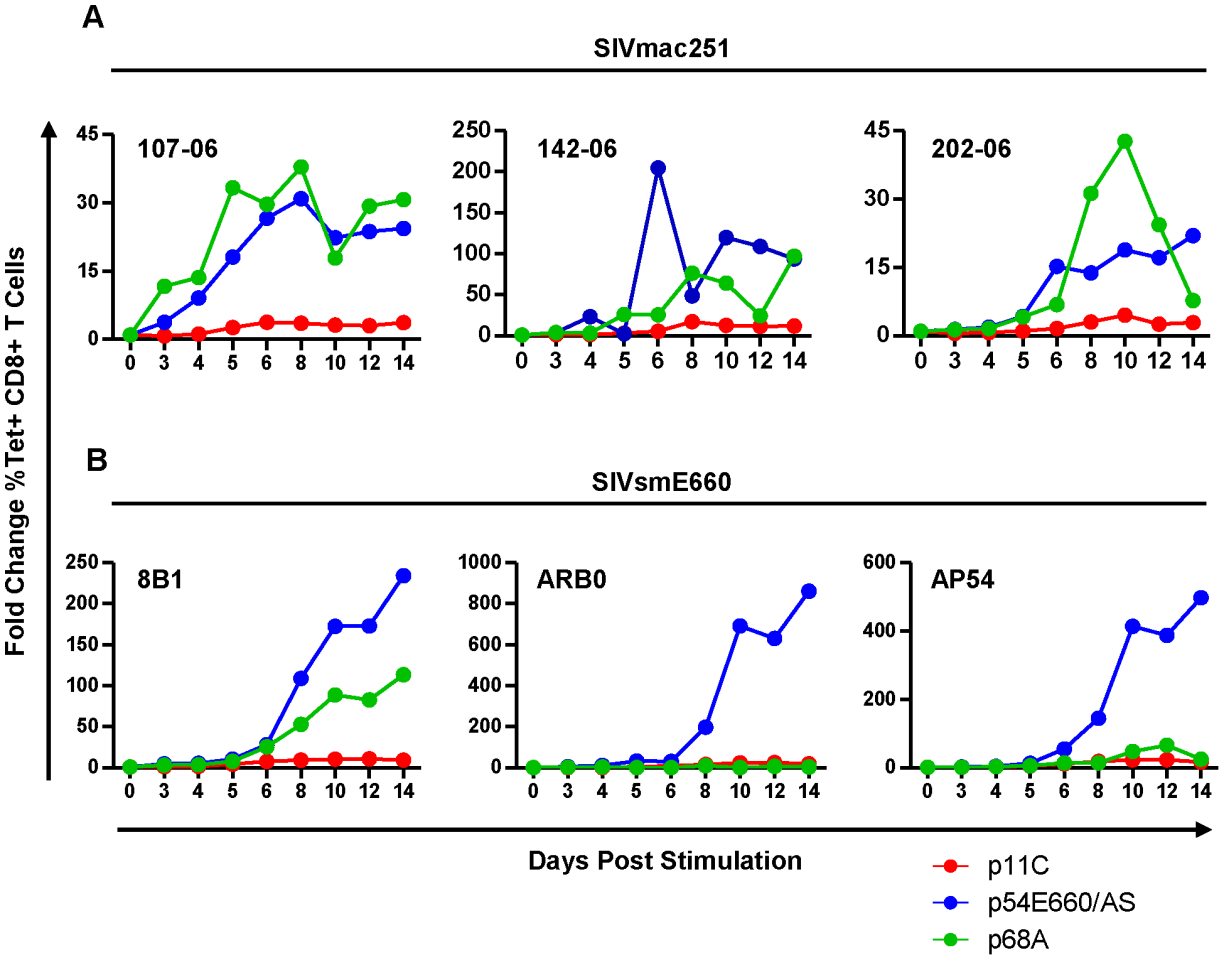
The dominant p11C-specific cells exhibited decreased antigen-specific expansion compared to subdominant epitope-specific cells. PBMCs from monkeys chronically-infected with either SIVmac251 (A) or SIVsmE660 (B) were stimulated *in vitro* with either p11C (red), p54E660/AS (blue), or p68A (green) peptide, harvested on days 3, 4, 5, 6, 8, 10, 12, and 14 following stimulation, and measured by flow cytometry to calculate the percent of tetramer-positive CD8^+^ T cells. Expansion was calculated as the fold change of the percent of each tetramer-positive population on each day, relative to day 0. Data from three SIVmac251- and three SIVsmE660-infected monkeys are shown. Measurements were conducted between weeks 40–52 for SIVmac251 and 31–44 for SIVsmE660.

Although differences in expression of genes involved in cell cycle and apoptosis were observed, it was unclear what impact these genes had on cell expansion upon antigen stimulation during chronic infection. To explore this, PBMCs from monkeys chronically infected with SIVmac251 or SIVsmE660 were peptide-stimulated and expansion of the epitope-specific cells was assessed over 14 days. We found that the dominant p11C-specific cells had reduced capacity to expand when compared to both of the subdominant epitope-specific cells (Figure 4 and Figure S6). This trend was consistent between cells from SIVsmE660- and SIVmac251-infected monkeys and is in agreement with prior reports that effector memory CD8^+^ T cells have limited proliferative capacity compared to less mature memory CD8^+^ T cells [41].

### The dominant p11C-specific CD8^+^ T cell population contained a lower frequency of cytokine- and chemokine-producing cells than the subdominant epitope-specific CD8^+^ T cell populations

**Figure 5:**
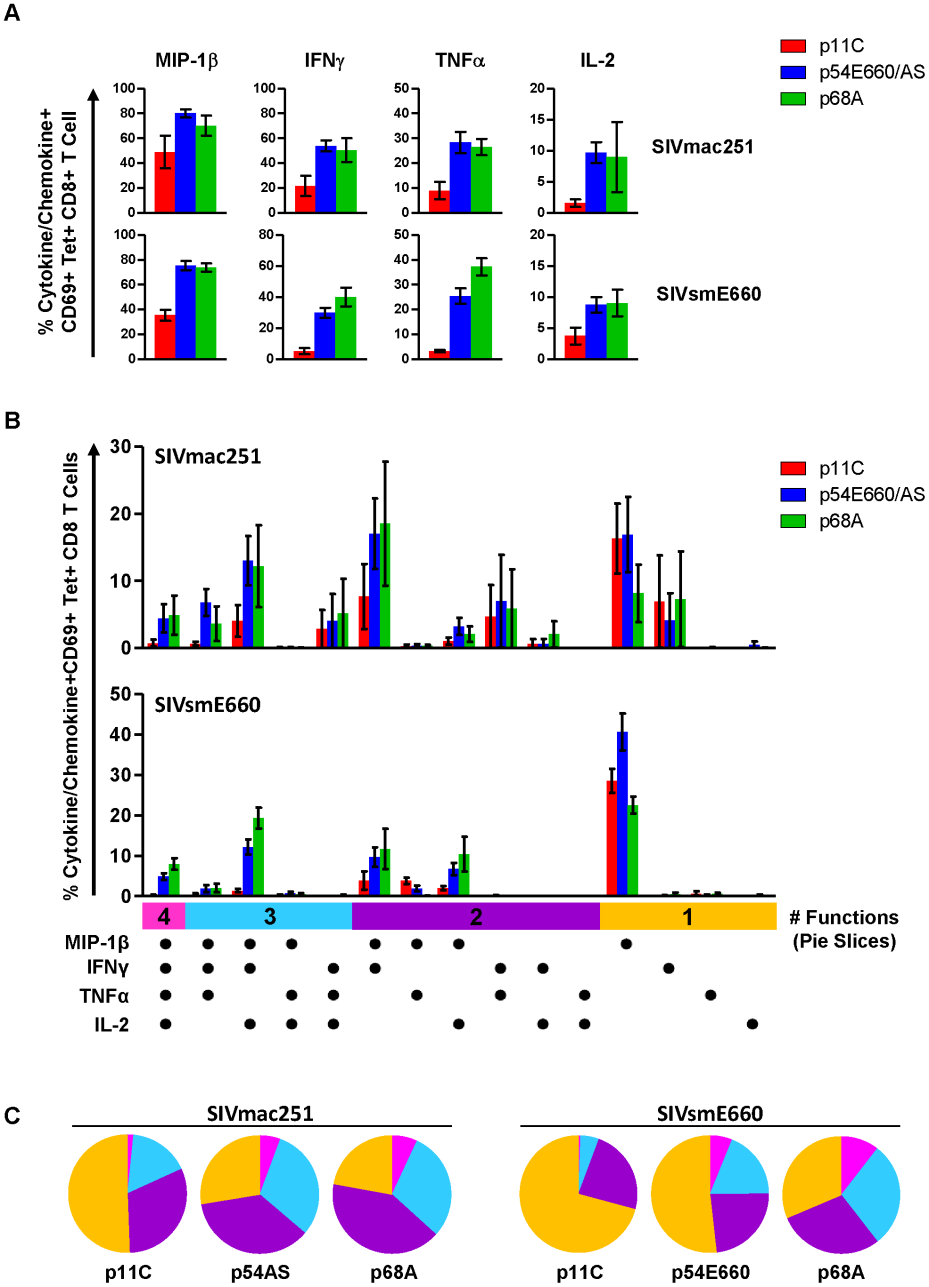
Dominant p11C-specific population contained lower frequency of cytokine- and chemokine-producing cells than subdominant epitope-specific populations. PBMCs from monkeys chronically-infected with either SIVmac251 (n = 4) or SIVsmE660 (n = 5) were stimulated with either p11C, p54AS/E660, or p68A peptides and intracellular staining was used to assess production of the chemokine MIP-1β and cytokines IFNγ, TNFα, and IL-2. Bars represent mean ± SEM. A) Individual cytokine and chemokine production. Top, SIVmac251. Bottom, SIVsmE660. B) Polyfunctional analysis. Positivity for each cytokine/chemokine is indicated by the dots below the bar graph. Vertical bars are grouped into 4, 3, 2 or 1 function (indicated by the pink, light blue, purple, and orange horizontal bars, respectively). C) Polyfunctionality charts. Left, SIVmac251. Right, SIVsmE660. Each slice represents the percentage of tetramer-positive cells expressing between 1 and 4 functions. Data were collected between weeks 36–42 for SIVmac251 and 14–25 for SIVsmE660.

Our gene expression data did not demonstrate differences in cytokine or chemokine expression between the p11C- and p54AS-specific cells. However, cytokine genes often require re-stimulation immediately prior to measurement [42]. Therefore, we considered that differences in cytokine expression between dominant and subdominant epitope-specific cells might be observed following antigen stimulation. To investigate, we stimulated PBMCs with epitope peptides and measured IL-2, TNFα, IFNγ, and MIP1-β production in standard intracellular cytokine staining (ICS) assays (Figure S7). Lower frequencies of cells expressing each of these cytokines and chemokines were present in the dominant p11C-specific cell population compared to the subdominant epitope-specific cell populations (Figure 5A). Furthermore, the subdominant epitope-specific populations were more polyfunctional, exhibiting larger proportions of cells expressing three or four functions, compared to the dominant p11C-specific population (Figure 5B and 5C).

### Expression of exhaustion-associated genes in dominant and subdominant CD8^+^ T cells

To assess differences in functionality that may be due to different extents of exhaustion, we compared the expression of genes encoding exhaustion-associated molecules between the p11C- and p54AS-specific cells during acute infection (day 14), at the onset of chronic infection (day 70), and during late chronic infection (week 31 or 32). The genes *LAG3* (NM_002286.4) and *CTLA4* (NM_005214.3) were significantly more highly expressed in the dominant p11C-specific cells during acute infection by 3.2- and 1.5-fold, respectively (Table 2). However, no genes were found differentially expressed on day 70. During late chronic infection, *LAG3* was expressed at increased levels in the dominant p11C-specific cells (1.7-fold). Therefore, the dominant p11C-specific cells showed higher expression of exhaustion-associated genes than the subdominant p54AS-specific cells.

**Table 2:** Expression of exhaustion-associated genes.

	Median Fold Change (p11C/p54AS)		
Gene (Protein)	Day 14	Day 70	Week 31/32
*cd244* (2B4)	−1.1	−1.1	1.0
*havcr2* (TIM-3)	−1.0	−1.1	1.1
*lag3* (LAG-3)	3.2*	1.0	1.7*
*pdcd1* (PD-1)	−1.1	−1.0	−1.1
*ctla4* (CTLA-4)	1.0	1.0	−1.0
*ctla4*	1.5*	1.0	−1.0
*ctla4*	−1.1	−1.1	1.0
*prdm1* (BLIMP-1)	1.1	1.1	1.1
*prdm1*	−1.0	−1.2	−1.0
*prdm1*	1.0	−1.1	−1.0
*prdm1*	−1.2	−1.1	−1.0
*cd160* (CD160)	−1.1	−1.2	−1.2
*tbx21* (T-bet)	1.1	−1.1	−1.1
*eomes* (Eomesodermin)	−1.0	−1.7	−1.1
*klrg1* (KLRG1)	−1.1	1.1	1.2
*klrg1*	1.1	−1.1	−1.0

* p≤0.05.

### High TCR:pMHC affinity correlates with the epitope dominance

We were interested in identifying mechanisms underlying the differences in the frequency and function of epitope-specific cells within an immunodominance hierarchy. Since TCR:pMHC interactions can influence T cell activation, proliferation, and function [43]–[49], we considered that TCR:pMHC interactions might play a role in the establishment of the magnitude or of the functionality of CD8^+^ T cells within an immunodominance hierarchy. We employed a surface plasmon resonance (SPR)-based technique developed in our laboratory that assesses the interaction of TCRs from a polyclonal epitope-specific CD8^+^ T cell population with monomeric pMHC complexes [50] to evaluate the role of TCR:pMHC binding in determining the Mamu-A*01-restricted epitope immunodominance hierarchy.

**Figure 6:**
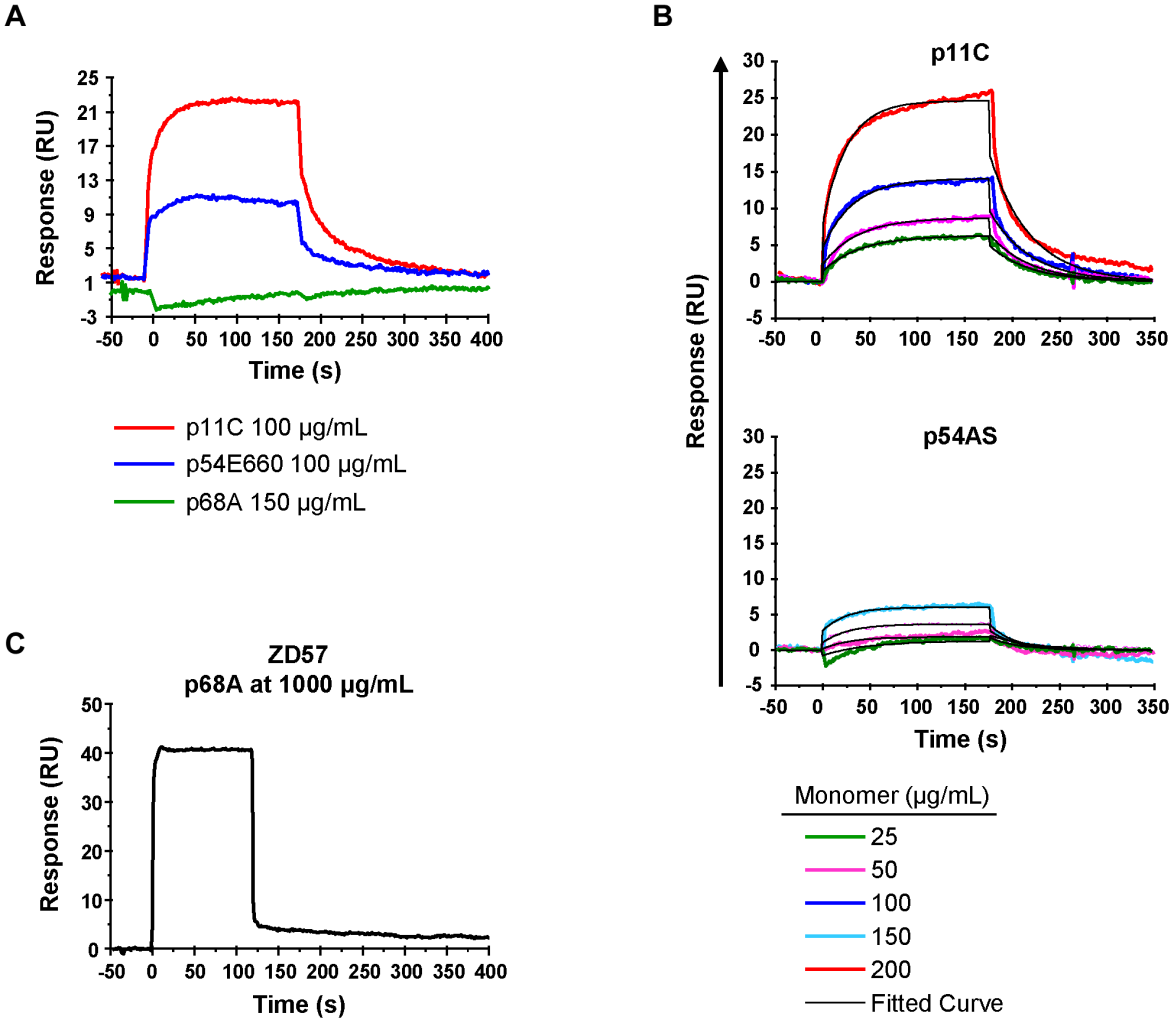
TCR:pMHC measurements. DRMs were purified from total CD8^+^ T cells sorted from seven chronically-infected SIVsmE660-infected monkeys. The DRMs were evaluated for specific binding, measured in resonance units (RU), to pMHC monomers constructed with p11C, p54E660, and p68A epitope peptides and Mamu-A*01. Data are representative of the binding observed from all seven monkeys evaluated. A) Initial experiments to detect p11C, p54E660, and p68A monomer binding to DRMs. 100 μg/mL p11C (red), 100 μg/mL p54E660 (blue), and 150 μg/mL p68A (green) pMHC monomer binding are overlaid from experiments conducted on separate Biacore Chips. Binding of the p68A:Mamu-A*01 monomer to the DRMs was not observed at any concentration of monomer tested (25–200 μg/mL). B) Titrations of p11C (top) and p54E660 (bottom) peptide:Mamu-A*01 monomers for calculation of binding kinetics and affinity. Overlaid sensograms of the binding of p11C and p54E660 pMHC monomers to DRMs purified from total CD8^+^ T cells are shown. Monomer binding was evaluated using pMHC concentrations ranging from 25 to 200 μg/mL. The black curve shows the Langmuir fitted curve that was used to calculate kinetics. C) Detection of p68A peptide:Mamu-A*01 monomer binding to DRMs. p68A-specific CD8^+^ T cells were collected from multiple tetramer-specific flow cytometric cell sorts and pooled for DRM purification. Titrations of p68A pMHC monomers were performed at concentrations ranging from 150 to 1000 μg/mL. Binding to DRMs from monkey ZD57 at 1000 μg/mL is shown and is representative of the four monkeys that were evaluated. All readings have been normalized by subtracting the binding of the control monomer TL8 run at the same concentrations as the experimental monomers.

For initial measurements of the binding of the p11C-, p54E660-, and p68A-specific TCRs to their respective monomeric pMHC complexes, we sorted total CD8^+^ T cells from seven Mamu-A*01^+^ SIVsmE660-infected monkeys. Detergent-resistant micro-domain (DRM) preparations from sorted cells, which were enriched in TCR complexes, were immobilized onto a Biacore L1 chip. Initial assays demonstrated that specific binding to DRM preparations could be detected using monomers constructed with the dominant p11C and subdominant p54E660 epitope peptides (Figure 6A and Figure S8). The binding signal from the p54E660 monomers was weaker than that of the p11C monomers at the same concentration. We were unable to detect specific binding of the monomers constructed with the more subdominant p68A epitope peptide at any of the concentrations of monomers (25–150 μg/mL) that were initially evaluated (Figure 6A).

We performed titrations of the p11C and p54E660 pMHC monomers on DRM preparations from all seven monkeys (Figure 6B and Figure S9) and used a 1∶1 Langmuir curve fitting analysis to calculate the association rate (*k_a_*) and the dissociation rate (*k_d_*) of the TCR:pMHC binding interaction (Table 3). The p11C:Mamu-A*01 monomers bound to the DRM samples with a median *k_on_* of 7.38×10^3^/Ms (range 3.56–28.6×10^3^/Ms), which was faster than the p54E660:Mamu-A*01 monomers that had a median *k_on_* of 0.96×10^3^/Ms (range 0.66–1.87×10^3^/Ms). The dissociation rate of p11C:Mamu-A*01 monomers from DRMs (median *k_off_* 0.024/s, range 0.015–0.030/s) was not substantially different from that of p54E660:Mamu-A*01 monomers (median *k_off_* of 0.033/s, range 0.021–0.061/s). The apparent dissociation constants (K_d_) were derived from the kinetic rate constant values and showed that the p11C:Mamu-A*01 monomers had a lower median K_d_ (2.0 μM, range 1.0–6.8 μM) than the p54E660:Mamu-A*01 monomers (32 μM, range 22–43 μM). Thus, monomers constructed with the dominant p11C peptide epitope exhibited faster association rates to chip-immobilized DRMs, resulting in higher affinities (lower K_d_) to TCRs than monomers constructed with the subdominant p54E660 epitope peptide.

**Table 3:** TCR:pMHC binding values.

	*k_on_* (×10^3^M^−1^s^−1^)	*k_off_* (s^−1^)	K_d_ (μM)
p11C	7.38 (3.56–28.6)	0.024 (0.015–0.030)	2.0 (1.0–6.8)
p54E660	0.96 (0.66–1.87)	0.033 (0.021–0.061)	32 (22–43)
p68A	≥10*	≥1‡	≥100†

Values are the median (range) from seven monkeys.

*k_on_*, association rate.

*k_off_*, dissociation rate.

K_d_, equilibrium dissociation constant. Calculated using the equation *k_off_*/*k_on_*.

* *k_on_* of the p68A monomers was estimated as being at least as fast as the fastest measured monomer, p11C.

‡ *k_off_* of p68A monomers was estimated as being at least as fast as the limit of detection of the Biacore instrument, approx.1/s.

† K_d_ of p68A monomer binding calculated using estimated values of the *k_on_* and *k_off_* using the equation *k_off_*/*k_on_*.

We speculated that the inability to detect the binding of p68A:Mamu-A*01 monomers to the DRM preparations may be due to a substantially lower affinity of the p68A:Mamu-A*01 complex for its cognate TCRs. The difficulty in detecting binding was also likely exacerbated by the low frequency of the p68A-specific cells, which would have resulted in underrepresentation of the p68A-specific TCRs in the DRMs. Therefore, we pooled DRMs from p68A-specific cells sorted from multiple time points. These samples contained two- to six-fold more p68A-specific cells than samples from the initial experiments.

Using these DRM preparations containing more p68A-specific cells and using higher concentrations of p68A:Mamu-A*01 monomers (>100 μg/mL), we detected specific DRM binding (Figure 6C and Figure S10). However, specific monomer binding exhibited extremely fast association and dissociation rates and was detected only at very high concentrations. As a result, reliable K_d_ values could not be measured using global curve fitting analyses. Our previous measurement of p11C and p54E660 pMHC monomer binding to DRMs indicated that p11C had the fastest association rate. The association rate of the p68A monomer to its respective TCRs was at least as fast as the association of the quantifiable p11C monomer, although likely to be even faster. Therefore, we estimated that the *k_on_* for p68A was at least 10×10^3^/Ms. Knowing that the Biacore instrument's lower limit of detection of dissociation is about 1/s, we estimated that the *k_off_* of the p68A:Mamu-A*01 monomer from its respective TCRs was at least as fast as 1/s. Using these estimated *k_on_* and *k_off_*, the apparent K_d_ of the p68A monomer was estimated be at least 100 μM (Table 3).

Therefore, the calculated K_d_ values were lowest for p11C monomers, intermediate for p54E660 monomers, and estimated to be highest for p68A monomers. In summary, epitope dominance was associated with the hierarchy of TCR affinities. Affinity differences were driven by the fast association rate of the dominant p11C epitope:Mamu-A*01 complex to its respective TCRs and likely fast dissociation rate of the more subdominant p68A:Mamu-A*01 complex.

## Discussion

The development of an effective vaccine against a pathogen such as HIV-1 will require a detailed understanding of the interplay between epitope immunodominance and the functional capacities of epitope-specific CD8^+^ T cells. The present study was undertaken to explore this relationship in SIV-infected rhesus monkeys.

It is important to note that our longitudinal gene expression analyses were conducted with a restricted sample size of six monkeys. In addition, because of the large numbers of cells required for RNA isolation, we were unable to perform phenotyping or functional assays during acute infection. Despite these clear limitations, we were able to detect a number of significant differences in gene expression between the dominant and subdominant epitope-specific populations during acute infection. Baron *et al* had similar findings, in a murine model, where they characterized a dominant (H7^a^) and a cryptic (HY) H2-D^b^–restricted epitope-specific CD8^+^ T cell population [20]. In the murine model, the cryptic epitope-specific cells had higher IL7R expression during primary expansion and higher L-selectin/SELL on all timepoints evaluated. Similarly, we found that both *IL7R* and *SELL* were higher in the subdominant p54AS-specific cells. Additionally, Baron *et al* found that *GZMA* was highly expressed in the dominant epitope-specific CD8^+^ T cells. We found a trend toward higher expression of *GZMA* and significantly higher expression of *GZMB* and *GZMK* in the dominant epitope-specific populations. This suggests that common gene networks are involved in the regulation of dominant immunologic responses.

We also evaluated the expression of exhaustion-associated genes between the dominant and subdominant epitope-specific cells. We found that the dominant p11C-specific cells had increased expression of genes encoding LAG-3 and CTLA-4 during acute infection, both of which are known to be upregulated upon TCR stimulation [51], [52]. The role of these exhaustion-associated genes during acute infection is not well established and it is unclear to what extent these molecules impair CD8^+^ T cell function during acute HIV/SIV infection. Their increased expression on the dominant p11C-specific cells during acute infection suggests that these cells were experiencing a greater level of activation driven by greater stimulation through their higher-affinity TCRs. On day 70, no differences in gene expression were noted between the dominant and subdominant epitope-specific cells. However, by late chronic infection, the dominant p11C-specific cells showed increased levels of *LAG-3* transcripts. LAG-3 is a well-described marker of exhausted CD8^+^ T cells during chronic viral infections [53], [54], including HIV [55]. However, while LAG-3 expression on CD8^+^ T cells has been shown to contribute to immune suppression in certain mouse models [56], its suppressive effect during HIV/SIV infection remains unclear [57], [58]. Additional studies are needed to confirm the cell surface expression of this protein and determine the extent to which increased expression contributes to functional differences between dominant and subdominant SIV/HIV epitope-specific cells.

In the present study, we found that dominant epitope-specific cells had greater cytotoxic potential, whereas the subdominant epitope-specific cells displayed enhanced proliferation capacity combined with increased cytokine and chemokine production. Importantly, these dominant epitope-specific cells had higher TCR affinity, a measure that typically correlates with functional avidity [59]–[61]. The ability of HIV/SIV epitopes with higher functional avidity to induce T cells that are less polyfunctional with lower proliferative capacity has been described by Harari *et al* [62] and Viganò *et al* [63]. These studies also corroborate our findings that cells of increased avidity also exhibited increased expression of exhaustion-associated molecules. Similarly, Conrad *et al* found that dominant TCR clonotypes exhibited reduced *in vitro* cytokine production and survival compared to the subdominant clonotypes that was associated with increased TCR avidity and increased expression of PD-1 [64]. These studies support the notion that reduced CD8^+^ T cell avidity promotes the development of polyfunctional T cells that under conditions of persistent high antigenemia may be able to partially control viral replication. This is also supported by the fact that increased avidity is significantly associated with viral escape [65]–[67] and increased induction of cell death and exhaustion [68], [69]. Collectively, these studies are consistent with our findings and suggest that to generate polyfunctional T cells after vaccination, the antigens used cannot confer extremely high TCR affinity; otherwise exhausted and monofunctional T cells will be generated.

The finding that the dominant p11C-specific cells were not highly polyfunctional is surprising considering that these cells are thought to be predominantly responsible for control of SIV viral replication [70], [71]. This reduced polyfunctionality may be compensated by a number of other cell characteristics. First, they may possess higher functional avidity, a characteristic that also correlates strongly with HIV control [15], [60], [72], [73] and higher TCR affinity [59]–[61]. Second, the dominant epitope-specific cells are present at substantially higher frequencies and contain greater amounts of perforin and granzyme than the subdominant epitope-specific cells. Thus on a percentage basis, the former population has an increased capacity to kill virus-infected cells. Finally, they may have greater TCR cross-reactivity to epitope variants, a characteristic that is thought to be an important component of durable HIV control by CD8^+^ T cells [73], [74]. In support of this possibility, Mothe *et al* found that HIV controllers had higher functional avidity that was associated with higher cross-reactivity of CD8^+^ T cells to HIV epitope variants compared to non-controllers [73]. Additionally, Bennett *et al* generated a panel of mutant TCRs specific for the HIV epitope SL9 and found that those TCR mutants with higher TCR avidities generally were more cross-reactive to SL9 variants than those with lower avidities [61]. In contrast, the previously-mentioned study by Conrad *et al* found that the high TCR avidity of the dominant clonotypes had reduced cross-reactivity than subdominant clonotypes [64]. Future studies should compare the TCR cross-reactivity to variant epitopes between the dominant p11C- and the subdominant epitope-specific cells to determine the extent that it contributes to the protective capacity of the p11C-specific cells.

We found that TCR affinity was predictive of frequency of the dominant and subdominant epitope-specific cells that are generated. The role of TCR:pMHC interactions in determining immunodominance hierarchies has been largely unexplored. A limited number of murine studies that have addressed this by measuring TCR dissociation rates using peptide:MHC class I tetramers have either found a lack of association between TCR affinity and epitope dominance [75] or found an association in the opposite direction [76]. The discordance between these studies and ours may be that for the measurement of the TCR dissociation rate the mouse studies used pMHC tetramers as opposed to monomers used in the present study. It is known that the accuracy of pMHC tetramers to predict physiologic TCR:pMHC binding affinities is limited [77], [78]; The multi-valency of tetramers combined with their propensity to aggregate can complicate accurate measurement of these interactions [79]. Moreover, the present study found that overall binding affinity (which incorporates both association and dissociation rate), rather than dissociation rate alone, was predictive of epitope dominance. In the above-mentioned murine studies, only the dissociation rate was assessed, leaving the association rate and affinity unknown. Slower association rates have been previously attributed to induced-fit or thermodynamic entropic penalty [80]–[82] in TCR binding and as such, pMHC epitopes that bind with faster association rates may outcompete those that bind with slower kinetic rates and gain dominance in response. Therefore, it is possible that association rate also played a role in the immunodominance hierarchies in these mouse studies and its measurement may have been informative about the particular parameter of the TCR:pMHC interactions that was important for immunodominance.

The present study found that high-frequency dominant epitope-specific CD8^+^ T cells had reduced proliferation and cytokine production but increased cytotoxicity potential compared to low-frequency subdominant epitope-specific CD8^+^ T cells. This was associated with higher TCR affinity and increased expression of exhaustion-associated genes in the dominant epitope-specific cells. The engineering of higher-affinity TCRs has been explored for enhancement of HIV immunity [61], [83]. However, our findings suggest that high TCR affinity may increase susceptibility to exhaustion and reduced functionality, although it may also promote higher cytotoxic potential. Since both proliferation and cytokine production as well as cytotoxicity are associated with superior control of HIV replication, antigen targets of engineered TCRs or antigens that are included in an HIV vaccine should be of both high and low affinity. The high-affinity antigens will promote epitope-specific CD8^+^ T cells of high frequency with high cytotoxic potential while the low-affinity antigens will promote CD8^+^ T cells that are more polyfunctional and proliferative and that have decreased potential for exhaustion.

## Materials and Methods

### Ethics statement

Indian-origin rhesus monkeys used in this study were maintained according to the guidelines of the National Institutes of Health (NIH) Guide to the Care and Use of Laboratory Animals and the approval of the Institutional Animal Care and Use Committee (IACUC) of Harvard Medical School (protocol # 03503) and the NIH. The institution also accepts as mandatory the Public Health Service Policy on Humane care and use of Laboratory Animals by Awardee Institutions and the NIH Principles for the Utilization and Care of Vertebrate Animals used in Testing, Research and Training. The New England Primate Research Center (NEPRC) is fully accredited by the Association for Assessment and Accreditation of Laboratory Animal Care International (AAALAC). The NEPRC has developed a comprehensive environmental enrichment, psychological well-being, and minimization of distress plans for primates that is available for inspection by the United States Animal and Plant Health Inspection Service (APHIS) and to officials of any pertinent funding agency. The Harvard Medical School IACUC documents NEPRC compliance with the plan during semiannual facility inspections.

### Animals

Mamu-A*01-positive, Mamu-B*17-negative, Mamu-B*08-negative, Indian-origin rhesus monkeys (*Macaca mulatta*) were selected for these studies by PCR-based MHC typing as previously described [84]. All monkeys were infected intrarectally with either SIVmac251 or SIVsmE660. SIVmac251 infection was administered by a single high dose challenge. SIVsmE660 was administered by a repeated low-dose challenge.

### Antibodies and flow cytometry reagents

Conjugated antibodies and staining reagents included MIP-1β-PE, CD3-PB, CD3-PE-Cy7, CD3-PerCP-Cy5.5, CD3-APC-Cy7, CD3-Horizon V450, CD4-PerCP-Cy5.5, CD4-AmCyan, CD4-FITC, CD8α-APC, CD8α-APC-Cy7, CD8α-AlexaFluor700, CD8α-FITC, CD8α-APC-H7, CD69- ECD (Beckman Coulter), CD20-Horizon V450, CCR7-FITC (R&D Systems), CD95-APC, CCR7-PerCP-Cy5.5, CD28-PE-Cy7 (eBioscience), granzyme B-AlexaFluor700, perforin-FITC (MabTech), IFNγ-PE-Cy7, TNFα- AlexaFluor700, IL-2-APC, CD95-PE, CD95-APC, and Aqua LIVE/DEAD Fixable Dead Cell Stain (Invitrogen). All reagents are from BD Biosciences unless indicated otherwise. For construction of monomers and tetramers, the following peptides were synthesized and purified to >95% by HPLC by New England Peptide LLC: p11C (CTPYDINQM), p54AS (TVPWPNASL), p54E660 (TVPWPNETL), p68A (STPPLVRLV), and TL8 (TTPESANL). The monomers and tetramers were prepared as previously described [85], [86]. Tetramers were prepared using either streptavidin-PE (Prozyme), -APC (Prozyme), -AlexaFluor488 (Invitrogen), or -Qdot655 (Invitrogen). Monomers used in surface plasmon resonance studies were further quantified using an *RC DC* protein kit (Bio-Rad).

### RNA extraction and microarray analysis

Peripheral blood was collected on days 14, 21, 28, 35, 42, 56, 70, and at week 31 or 32 post-inoculation with SIVmac251. Plasma viral RNA from these samples was measured using an ultra-sensitive branched DNA amplification assay (Siemens Diagnostics, Berkeley, CA). PBMC were stained with p11C and p54AS tetramers and CD3 and CD8 antibodies at 4°C. Tetramer-positive single CD3^+^CD8^+^ lymphocytes were sorted to ≥95% purity into RNAprotect (Qiagen) at 4°C. RNA was isolated using a Trizol (Invitrogen) extraction protocol. Briefly, 0.8 ml of Trizol were added to the cell pellet and incubated for 5 minutes at room temperature and 0.16 ml of chloroform were added, shaken vigorously by hand for 15 seconds, incubated at room temperature for 2–3 minutes and centrifuged at 13,000 rpm for 15 minutes at 4°C. The colorless upper aqueous phase was carefully collected and transferred to a new tube containing 2 μl of linear acrylamide for mixing. An equal volume of isopropyl alcohol was then added and mixed. The mixture was incubated at room temperature for 10 minutes and centrifuged at 13,000 rpm for 10 minutes at 4°C. The supernatant was collected and the RNA was washed with 1 ml of 70% ethanol and centrifuged at 10,500 rpm for 5 minutes at 4°C. The supernatant was completely removed and the RNA pellet was allowed to air-dry. The RNA was then resuspended in RNase-free water and stored at −80°C. RNA integrity was tested using an Agilent Bioanalyzer. RNA was then amplified using the TargetAmp 2-Round Biotin-aRNA Amplification Kit 3.0 (Epicentre Biotechnologies) according the manufacturer's instructions. Amplified biotinylated antisense-RNA (aRNA) was resuspended in RNase-free water and stored at −80°C. Nanodrop ND-1000 was used to determine the biotinylated aRNA concentration and an Agilent Bioanalyzer was used to determine its integrity. Amplified aRNA was hybridized to Illumina Human HT-12 Expression BeadChips according to the manufacturer's instructions and was stained with Streptavidin Cy3 for detection (Illumina, San Diego, CA, USA). The Human HT-12 BeadChip assays 48,000 transcripts. The BeadChips were built with sequences derived from the National Center for Biotechnology Information Reference Sequence (NCBI RefSeq) database (Build 36.2, Release 22). Arrays were scanned according to the manufacturer's instructions. Processing of the raw array data was performed using Illumina BeadStudio software. Matlab (Mathworks, Natick, MA, USA) was used to perform statistical analysis of gene expression data. The entire expression dataset was first quantile-normalized. Fold-differences of individual transcripts were computed as the ratios of the median expression levels of the p11C- to those of the p54AS-specific CD8^+^ T cells (p11C/p54AS). When p54AS expression values were larger than p11C values, the negative reciprocal was calculated. A Wilcoxon signed-rank test was used to evaluate the significance of the differences in gene expression between p11C- and p54AS-specific CD8^+^ T cells. Genes whose expression was significantly different between the p11C- and p54AS-specific cell samples and whose median fold change difference was at least ±1.5 were considered differentially expressed. The Minimum Information about a Microarray Experiment (MIAME) criteria have been met by these experiments. The complete dataset is available in the Gene Expression Omnibus, accession number GSE54001.

### Flow cytometric analysis

In order to collect and analyze comparable numbers of each CD8^+^ T cell specificity, different numbers of PBMCs were used for each p11C, p54, and p68A sample. Input numbers of PBMCs were dependent on the expected relative frequency of each epitope-specific population. For example, if the expected frequencies of the p11C-, p54-, and p68A-specific CD8^+^ T cells were 10%, 1%, and 0.1%, respectively, then the ratio of the numbers of cells used for each sample was 1∶10∶100. Staining volumes, washing volumes, and amounts of staining reagents used were adjusted for each sample to ensure the same cell-to-reagent concentration ratios were used for all samples. For quantification of epitope-specific CD8^+^ T cells, PBMCs were stained with tetramer and CD3, CD4, and CD8 antibodies. For phenotyping, PBMCs were additionally stained with CD20, CD95, CD28, and CCR7 antibodies. For *ex vivo* measurement of perforin and granzyme B, PBMCs were stained with tetramer, CD3, CD4, and CD8 antibodies and then stained intracellularly with perforin and granzyme B antibodies as previously described [87]. FMO samples were also prepared to set the gates for perforin and granzyme B gating. All flow cytometric data were collected on an LSRII (BD) and analyzed using FlowJo (Tree star). Statistical analysis of flow cytometric data was conducted in GraphPad Prism 5. Wilcoxon signed rank test was used to compare groups. *P-values* ≤0.05 were considered significant. Bonferroni correction was used for multiple comparisons with significant *p-values* ≤0.05/(number of comparisons) considered significant.

### Cytokine and chemokine production

Measurements of IL-2, IFNγ, TNFα, and MIP-1β production by epitope-specific CD8^+^ T cells following stimulation of 1 nM peptide was performed in 5 mL FACS tubes as previously described [50]. Flow cytometric data were gated on single, live, CD3^+^CD4^−^CD8^+^CD69^+^ lymphocytes. Boolean gating was used to calculate percent of polyfunctional cells.

### Expansion of epitope-specific CD8^+^ T cells

PBMCs, at approximately 3×10^6^ lymphocytes/mL (measured using Guava EasyCyte automatic cytometer (Millipore)), were plated in flat-bottomed culture plates and stimulated for expansion with 1 nM epitope peptide. Approximately 1×10^6^ PBMCs were also stained with tetramers and CD3, CD4, and CD8 antibodies, to measure the frequency each CD8^+^ T cell specificity before peptide-stimulated expansion (day 0). 20 U/mL IL-2 (Hoffmann-LaRoche) was added to stimulated cells on day 3, and media supplemented with IL-2 was changed thereafter as needed. Samples were harvested on days 3, 4, 5, 6, 8, 10, 12, and 14 post-stimulation for staining with aqua LIVE/DEAD, tetramer and CD3, CD4, and CD8 antibodies for flow cytometric analysis. Flow cytometric data were gated on single, live, CD3^+^CD4^−^CD8^+^ tet^+^ lymphocytes.

### Surface plasmon resonance (SPR) measurements

Freshly isolated PBMCs were enriched for CD8^+^ T lymphocytes using Miltenyi's magnetic-activated cell sorting (MACS) kit for isolation of untouched NHP CD8^+^ T cells according to the manufacturer's instructions. Isolated CD8^+^ T cells were then stained at 4°C with tetramers and CD3 and CD8 antibodies. Sorting was performed on an Aria flow cytometer/cell sorter (BD). For initial TCR binding analyses, cells were only stained with the TL8 tetramers and those cells that were positive for TL8 were excluded from the sorted CD3^+^CD8^+^ lymphocytes. For repeated TCR binding analysis for enrichment of p68A-specific TCRs, cells were stained with TL8, p54E660, and p68A tetramers, and multiple populations were simultaneously sorted: p54E660^−^ p68A^−^ TL8^−^, p54E660^+^ p68A^−^ TL8^−^, and p54E660^−^ p68A^+^ TL8^−^. These sorted populations were subsequently combined during purification of the DRMs. For samples with low total cell number, extra DRMs from cells not specific for any of the epitopes being evaluated were added to provide extra mass to the pellet during centrifugation. These extra DRMs were obtained from sorted single CD3^+^CD8^+^ lymphocytes that were negative for all evaluated epitope specificities (p11C^−^p54E660^−^p68A^−^TL8^−^). All sorted cells were resuspended in a 4°C solution of protease inhibitors (1 μg/mL each leupeptin, pepstatin, and aprotinin) and stored at −80°C until analysis. DRM purification was carried out as described previously [50] with the modification that the repeat samples for measurements of p68A were conducted at concentrations up to 1,000 μg/mL of monomer. The specific binding signal was obtained by subtracting the non-specific signal from TL8 pMHC control monomer binding to the TL8-depleted DRM preparation when injected at the same concentration from the p11C, p54E660, or p68A binding signal. The global curve fitting to the Langmuir equation was used to derive kinetic rate constants (*k_on_* and *k_off_*) for calculation of equilibrium dissociation constant K_d_ as previously described [88], [89]. All SPR measurements were carried out on a Biacore 3000 instrument, and data analyses were performed using BIAevaluation 4.1 software (GE Healthcare).

## Supporting Information

Figure S1 **Gating of p11C- and p54AS-specific CD8^+^ T cells sorted for microarray analyses.** Gates for p11C (y-axis) and p54AS (x-axis) tetramers on samples sorted from each of the six Mamu-A*01 ^+^ rhesus monkeys (107-06, 112-06, 125-06, 133-06, 142-06, and 202-06) used in the gene expression analysis. Plots are gated on single CD3^+^CD8^+^ lymphocytes and percent of tetramer-positive cells are shown on each plot for the p11C- (upper left) and p54AS- (middle right) positive cells. 50,000 CD8^+^ T cells events are displayed for all samples except where indicated in upper right corner of plots. The data file for 107-06 on day 56 was corrupt and not available for re-analysis.
(TIF)

Figure S2 **Individual values of genes differentially expressed between dominant p11C- and subdominant p54AS-specific CD8^+^ T cells.** Shown are the individual normalized expression, measured in fluorescence units, for each A) maturation, B) cytotoxicity, and C) proliferation and apoptosis gene that was determined to be differentially expressed between p11C- and p54AS-specific cells. Plots include values for which its matching pair is missing and therefore were not used in determination of differential expression. Fold change values are indicated in upper left corner. An asterisk under fold change values is present if that gene met the criteria for differential expression on that timepoint. For those genes with more than one probe on the BeadChip, the numerical probe IDs are included in the gene name. Red, p11C. Blue, p54AS.
(PDF)

Figure S3 Staining and gating of tetramers for quantification of epitope-specific cells in Mamu-A*01 immunodominance hierarchies. Gating of p11C-, p54AS/E660-, and p68A-specific CD8^+^ T cells from A) SIVmac251- and B) SIVsmE660-infected animals. 50,000 CD8^+^ T cell events are displayed on each flow plot. SIVmac251 frequencies were measured between weeks 37–50, except for 133-06 which died early and data used are from week 18. SIVsmE660 frequencies were measured between weeks 19–22.
(TIF)

Figure S4 **Representative flow staining and gating for phenotyping.** Representative staining and gating for phenotyping of tetramer-positive cells from (A) SIVmac251- and (B) SIVsmE660-infected monkeys. Left, Staining and gating of tetramer-positive cells among CD8^+^ T cells (40,000 CD8^+^ T cell events are shown). Right, staining and gating of CD28 (y-axis) and CCR7 (x-axis) cell surface proteins among the tetramer-positive cells (1,000 tetramer-positive events are shown). Phenotyping was conducted between weeks 44–78 for SIVmac251 and 33–46 for SIVsmE660.
(TIF)

Figure S5 **Representative staining and gating of perforin and granzyme B.** Mamu-A*01 tetramers were used to identify p11C-, p54AS/E660-, and p68A-specific cells among PBMCs from monkeys chronically-infected with either SIVmac251 or SIVsmE660. Perforin and granzyme B content within the tetramer-positive cells were measured by flow cytometry. A) FMOs used to set gates for perforin (left) and granzyme B (right). For SIVmac251 (B) and SIVsmE660 (D), representative staining and gating of tetramer-positive cells (left) and perforin^+^granzyme B^+^ cells among the tetramer-positive cells (right). 250,000 CD8^+^ T cells are shown on flow plots of tetramer staining. 1,000 tetramer-positive events are shown on flow plots of perforin and granzyme B staining. For SIVmac251 (C) and SIVsmE660 (E), histograms illustrating fluorescence intensity of perforin (top) and granzyme B (bottom) within the p11C (red), p54AS/E660 (blue), and p68A (green) tetramer-positive cells. FMOs shown as filled gray histograms. Measurements were conducted between weeks 63–83 for SIVmac251 and 41–49 for SIVsmE660.
(TIF)

Figure S6 **Representative staining and gating of tetramers during** ***in vitro*** **expansion.** PBMCs from monkeys chronically-infected with either SIVmac251 (A) or SIVsmE660 (B) were stimulated *in vitro* with either p11C, 54E660/AS, or p68A peptide, harvested on days 3, 4, 5, 6, 8, 10, 12, and 14 following stimulation, and measured by flow cytometry to calculate the percent (upper right on each flow plot) of tetramer-positive CD8^+^ T cells. Expansion was calculated as the fold change (middle left on each flow plot) of the percent of each tetramer-positive population on each day, relative to day 0. Number of CD8^+^ T cell events displayed are the same for pllC, p54AS/E660, and p68A plots within a given day. Measurements were conducted between weeks 40–52 for SIVmac251 and 31–44 for SIVsmE660.
(TIF)

Figure S7 **Representative staining and gating for cytokine and chemokine production.** PBMCs from monkeys chronically-infected with either SIVmac251 (A) or SIVsmE660 (B) were stimulated with either p11C, p54AS/E660, or p68A peptides and intracellular staining was used to assess production of the chemokine MIP-1β and the cytokines IFNγ, TNFα, and IL-2. Left, representative staining and gating of tetramer-positive cells (25,000 CD8^+^ T cell events are shown). Right, corresponding staining and gating of MIP-1β, IFNγ, TNFα, and IL-2 (250 tetramer-positive events are shown). Individual gates were then used in a Boolean analysis for assessment of polyfunctionality. Data was collected between weeks 36–42 for SIVmac251 and 14–25 for SIVsmE660.
(PDF)

Figure S8 Detection of specific binding of p11C and p54E660 peptide:Mamu-A*01 monomers to DRMs. DRMs were purified from total CD8^+^ T cells sorted from seven chronically-infected SIVsmE660-infected monkeys. The DRMs were evaluated for specific binding, measured in resonance units (RU), to pMHC monomers constructed with p11C, p54E660, and p68A epitope peptides and Mamu-A*01. Shown are overlaid readings of the binding of p11C (red) and p54E660 (blue) pMHC monomers at 100 μg/mL. p68A:Mamu-A*01 monomer binding above background was not detected at any concentration and is not shown. Readings have been normalized by subtracting the binding of the control monomer TL8 run at the same concentrations as the experimental monomers.
(TIF)

Figure S9 Titrations of p11C and p54E660 peptide:Mamu-A*01 monomers for calculation of binding kinetics and affinity. Shown are sensograms indicating the binding of p11C (left) and p54E660 (right) pMHC monomers to DRMs purified from total CD8^+^ T cells sorted from seven SIVsmE660-infected monkeys. p11C monomers were run at 25 (green), 50 (pink), 100 (blue), and 200 (red) μg/mL. The ARB0 plot for p11C shows a 150 μg/mL (light blue) run in place of the 100 μg/mL. The AP34, ZD57, and A6V031 plots for p11C do not show the 200 μg/mL run. p54E660 monomers were run at 25, 50, 150, and 200 μg/mL for AP54, ARB0, 8B1, and AS47 and at 25, 100, and 200 μg/mL for AP34, ZD57, and A6V031. The ZD57 plot includes an additional 50 μg/mL run. A Langmuir curve was fit to each binding curve at each concentration and was used to calculate binding kinetics. Readings have been normalized by subtracting the binding of the control monomer TL8 run at the same concentrations as the experimental monomers.
(PDF)

Figure S10 **Detection of p68A monomer binding.** p68A-specific CD8^+^ T cells were collected from multiple tetramer-specific flow cytometric cell sorts and pooled for DRM purification. Titrations of p68A pMHC monomers were performed at concentrations ranging from 150 to 1000 μg/mL. The highest concentration evaluated is shown. Binding of the control monomer TL8 at the same concentration has been subtracted from all readings.
(TIF)
