# Supplementary figures and images for: TCR Affinity Associated with Functional Differences between Dominant and Subdominant SIV Epitope-Specific CD8+ T Cells in Mamu-A*01 + Rhesus Monkeys
Source: PLoS Pathog. 2014 Apr 17;10(4):e1004069. doi: 10.1371/journal.ppat.1004069 (PMC3990730; doi:10.1371/journal.ppat.1004069)

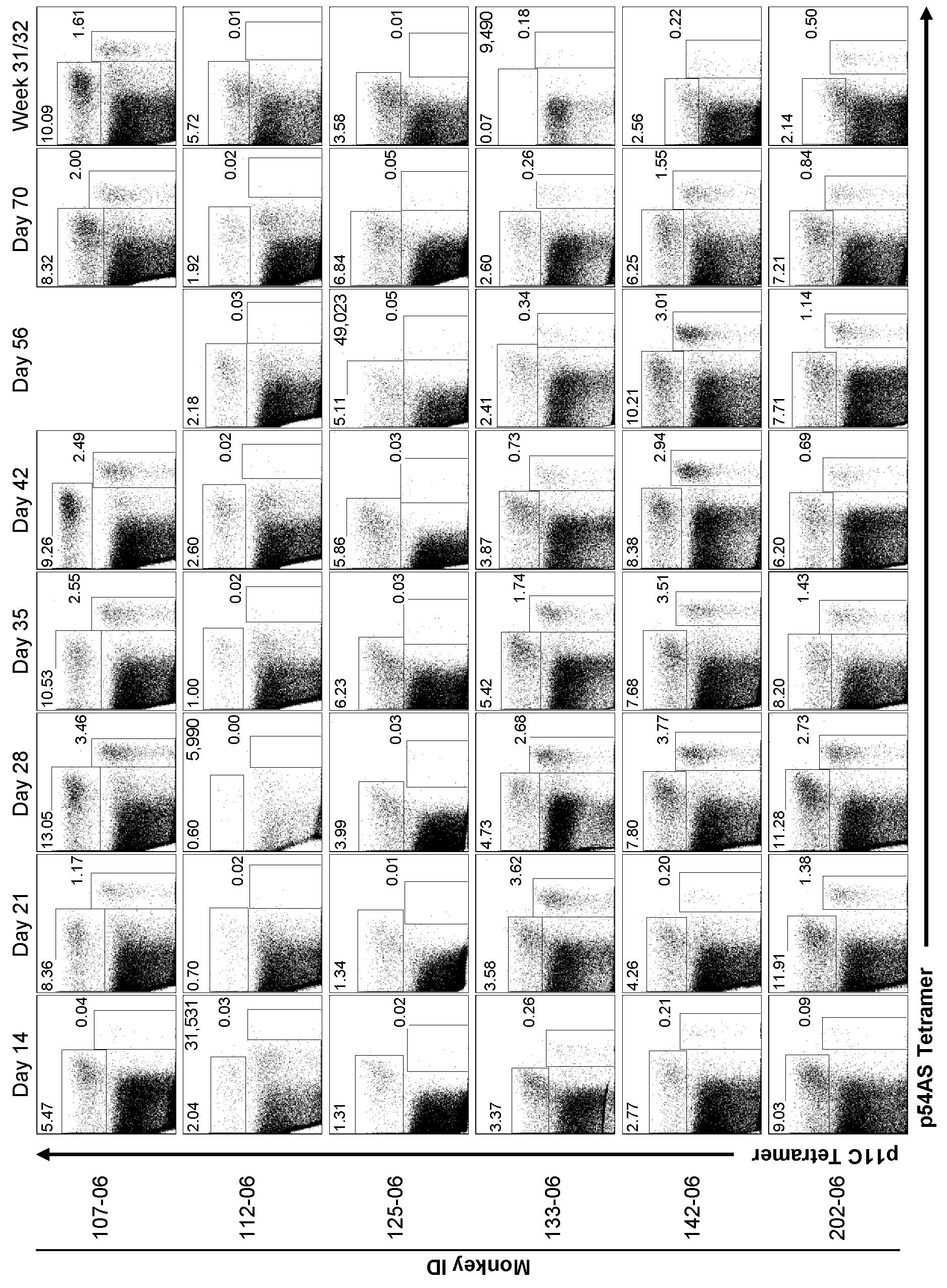

Supplement: Figure S1 — Gating of p11C- and p54AS-specific CD8+ T cells sorted for microarray analyses. Gates for p11C (y-axis) and p54AS (x-axis) tetramers on samples sorted from each of the six Mamu-A*01 + rhesus monkeys (107-06, 112-06, 125-06, 133-06, 142-06, and 202-06) used in the gene expression analysis. Plots are gated on single CD3+CD8+ lymphocytes and percent of tetramer-positive cells are shown on each plot for the p11C- (upper left) and p54AS- (middle right) positive cells. 50,000 CD8+ T cells events are displayed for all samples except where indicated in upper right corner of plots. The data file for 107-06 on day 56 was corrupt and not available for re-analysis. (TIF) [file ppat.1004069.s001.tif]

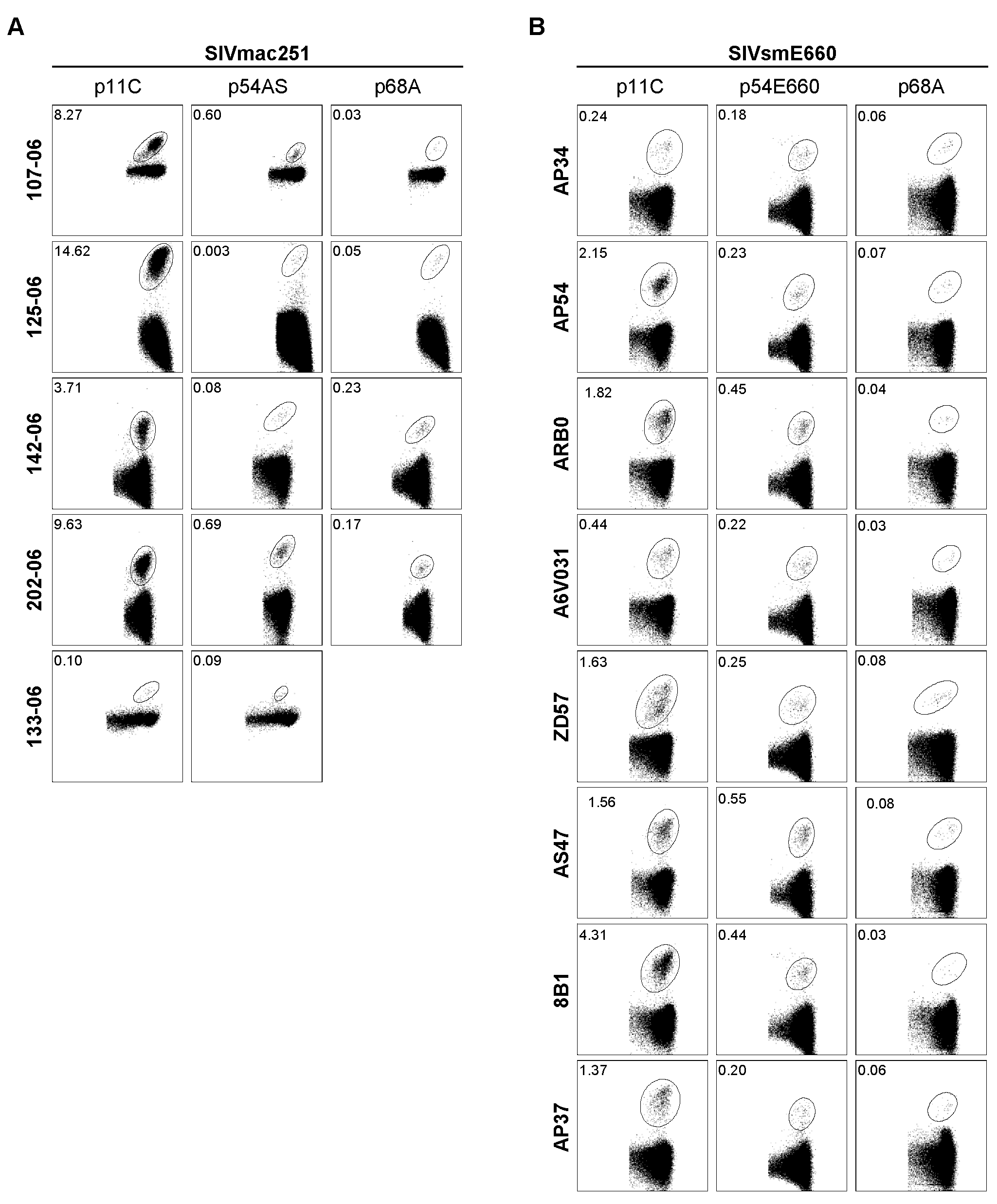

Supplement: Figure S3 — Staining and gating of tetramers for quantification of epitope-specific cells in Mamu-A*01 immunodominance hierarchies. Gating of p11C-, p54AS/E660-, and p68A-specific CD8+ T cells from A) SIVmac251- and B) SIVsmE660-infected animals. 50,000 CD8+ T cell events are displayed on each flow plot. SIVmac251 frequencies were measured between weeks 37–50, except for 133-06 which died early and data used are from week 18. SIVsmE660 frequencies were measured between weeks 19–22. (TIF) [file ppat.1004069.s003.tif]

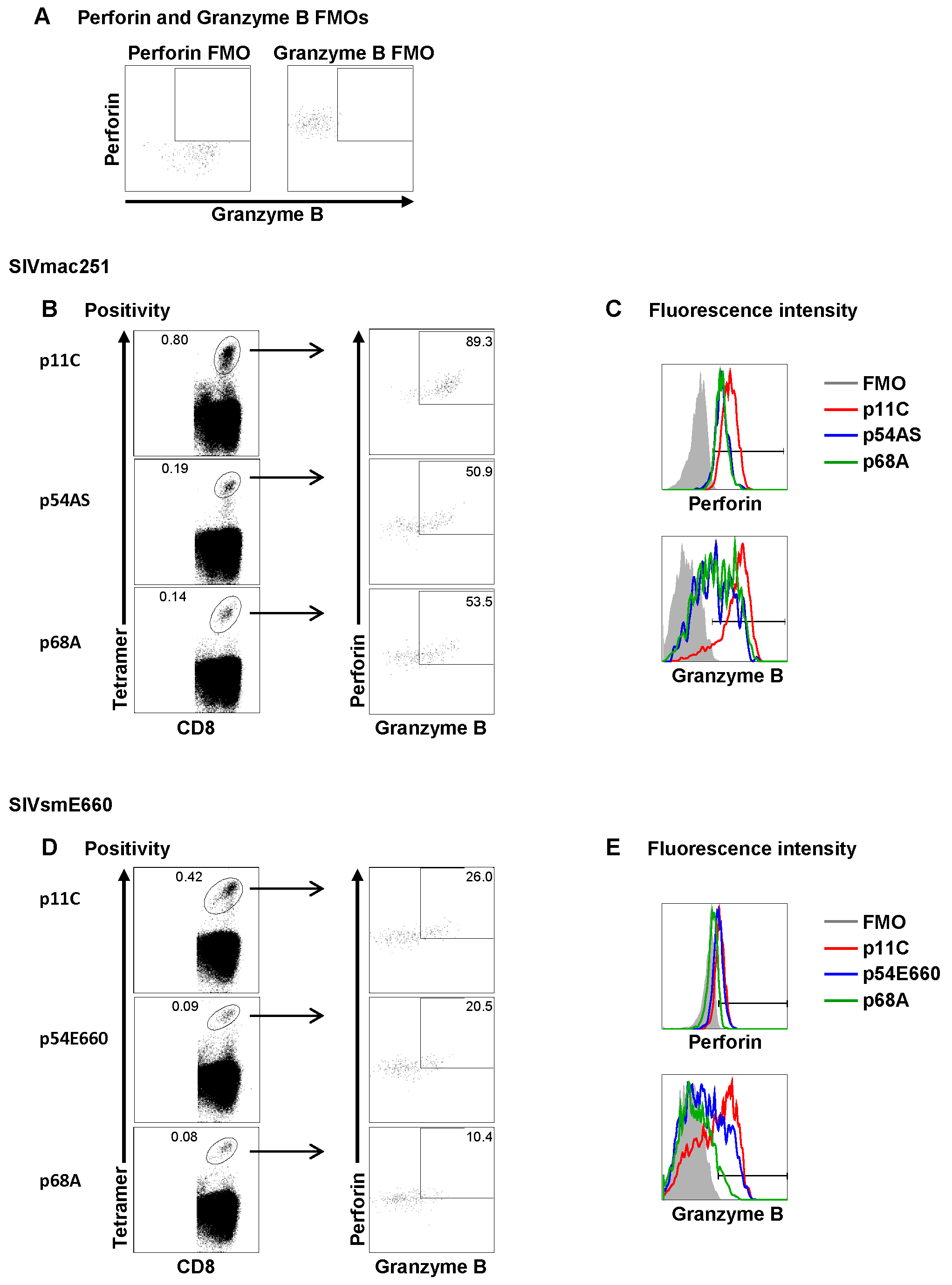

Supplement: Figure S5 — Representative staining and gating of perforin and granzyme B. Mamu-A*01 tetramers were used to identify p11C-, p54AS/E660-, and p68A-specific cells among PBMCs from monkeys chronically-infected with either SIVmac251 or SIVsmE660. Perforin and granzyme B content within the tetramer-positive cells were measured by flow cytometry. A) FMOs used to set gates for perforin (left) and granzyme B (right). For SIVmac251 (B) and SIVsmE660 (D), representative staining and gating of tetramer-positive cells (left) and perforin+granzyme B+ cells among the tetramer-positive cells (right). 250,000 CD8+ T cells are shown on flow plots of tetramer staining. 1,000 tetramer-positive events are shown on flow plots of perforin and granzyme B staining. For SIVmac251 (C) and SIVsmE660 (E), histograms illustrating fluorescence intensity of perforin (top) and granzyme B (bottom) within the p11C (red), p54AS/E660 (blue), and p68A (green) tetramer-positive cells. FMOs shown as filled gray histograms. Measurements were conducted between weeks 63–83 for SIVmac251 and 41–49 for SIVsmE660. (TIF) [file ppat.1004069.s005.tif]

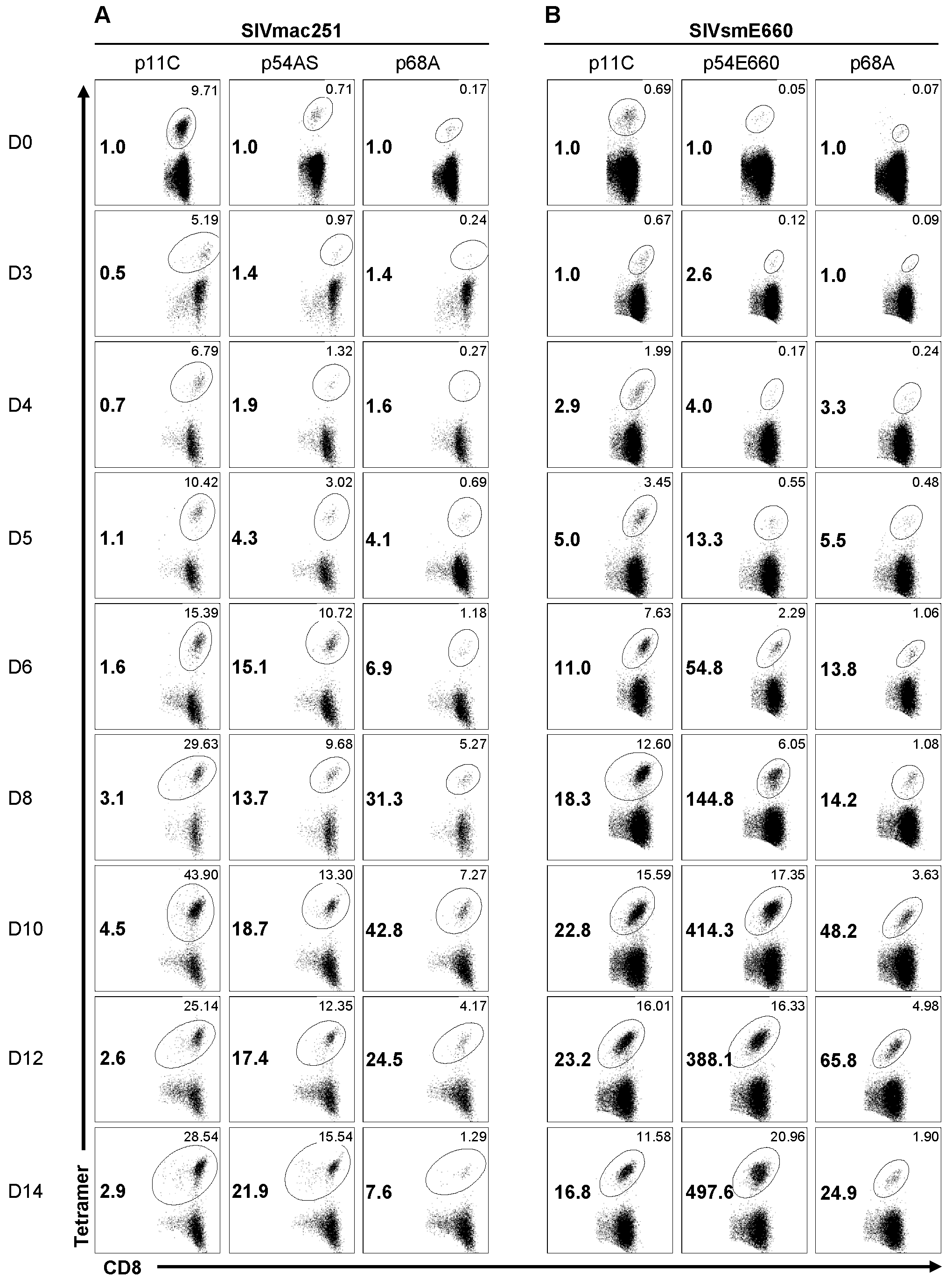

Supplement: Figure S6 — Representative staining and gating of tetramers during in vitro expansion. PBMCs from monkeys chronically-infected with either SIVmac251 (A) or SIVsmE660 (B) were stimulated in vitro with either p11C, 54E660/AS, or p68A peptide, harvested on days 3, 4, 5, 6, 8, 10, 12, and 14 following stimulation, and measured by flow cytometry to calculate the percent (upper right on each flow plot) of tetramer-positive CD8+ T cells. Expansion was calculated as the fold change (middle left on each flow plot) of the percent of each tetramer-positive population on each day, relative to day 0. Number of CD8+ T cell events displayed are the same for pllC, p54AS/E660, and p68A plots within a given day. Measurements were conducted between weeks 40–52 for SIVmac251 and 31–44 for SIVsmE660. (TIF) [file ppat.1004069.s006.tif]

## A SIVmac251

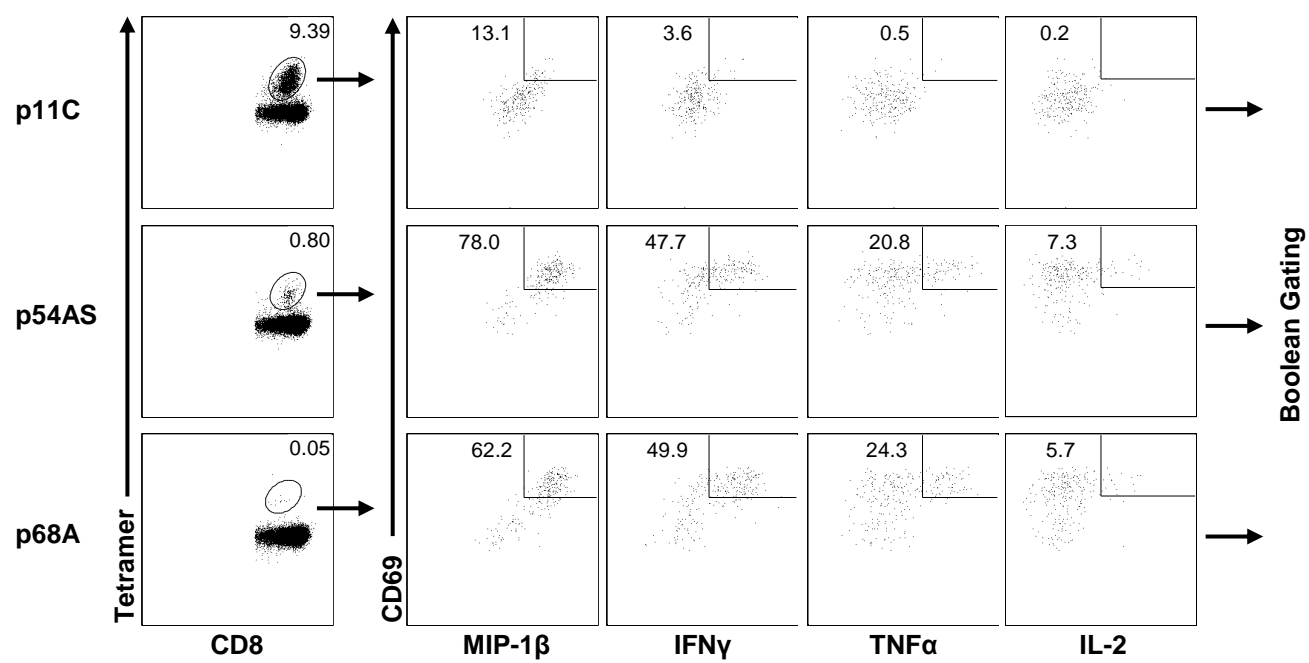

## B SIVsmE660

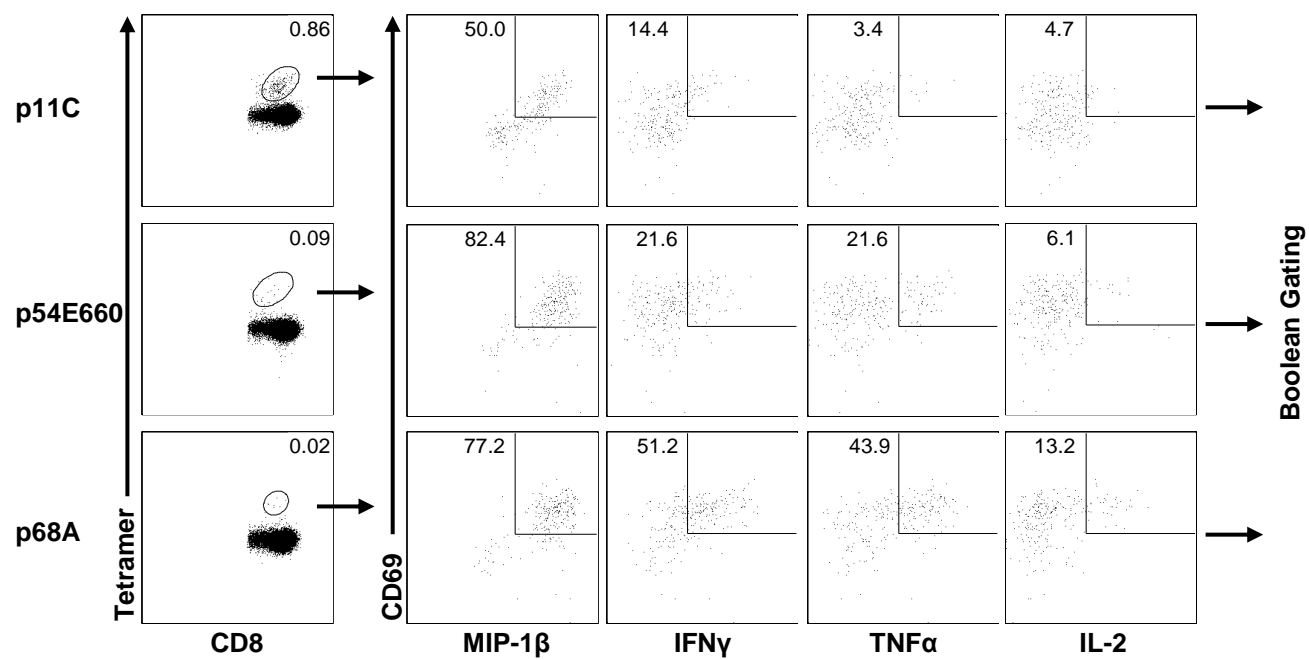

Supplement: Figure S7 — Representative staining and gating for cytokine and chemokine production. PBMCs from monkeys chronically-infected with either SIVmac251 (A) or SIVsmE660 (B) were stimulated with either p11C, p54AS/E660, or p68A peptides and intracellular staining was used to assess production of the chemokine MIP-1β and the cytokines IFNγ, TNFα, and IL-2. Left, representative staining and gating of tetramer-positive cells (25,000 CD8+ T cell events are shown). Right, corresponding staining and gating of MIP-1β, IFNγ, TNFα, and IL-2 (250 tetramer-positive events are shown). Individual gates were then used in a Boolean analysis for assessment of polyfunctionality. Data was collected between weeks 36–42 for SIVmac251 and 14–25 for SIVsmE660. (PDF) [file ppat.1004069.s007.pdf]

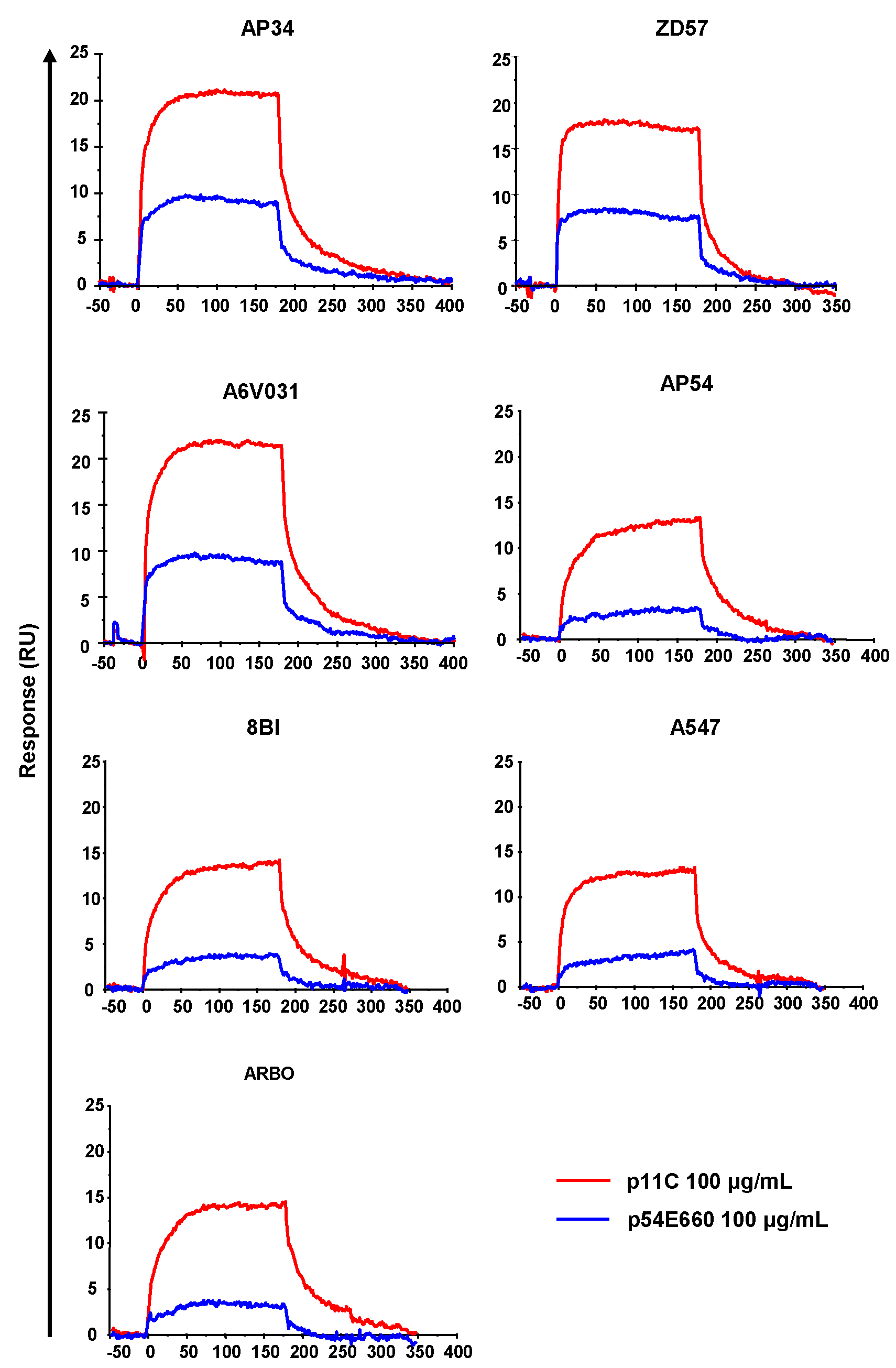

Supplement: Figure S8 — Detection of specific binding of p11C and p54E660 peptide:Mamu-A*01 monomers to DRMs. DRMs were purified from total CD8+ T cells sorted from seven chronically-infected SIVsmE660-infected monkeys. The DRMs were evaluated for specific binding, measured in resonance units (RU), to pMHC monomers constructed with p11C, p54E660, and p68A epitope peptides and Mamu-A*01. Shown are overlaid readings of the binding of p11C (red) and p54E660 (blue) pMHC monomers at 100 μg/mL. p68A:Mamu-A*01 monomer binding above background was not detected at any concentration and is not shown. Readings have been normalized by subtracting the binding of the control monomer TL8 run at the same concentrations as the experimental monomers. (TIF) [file ppat.1004069.s008.tif]

Response (RU)

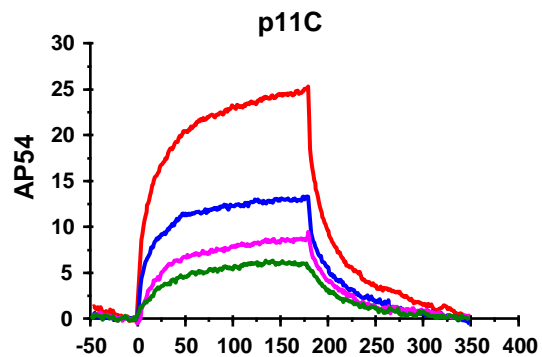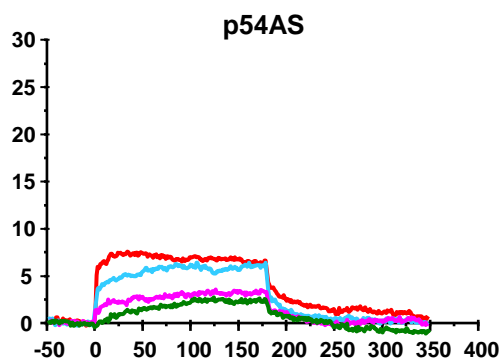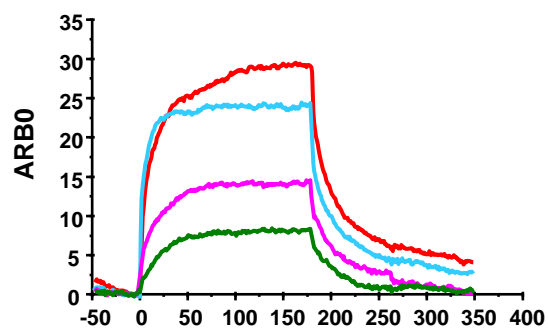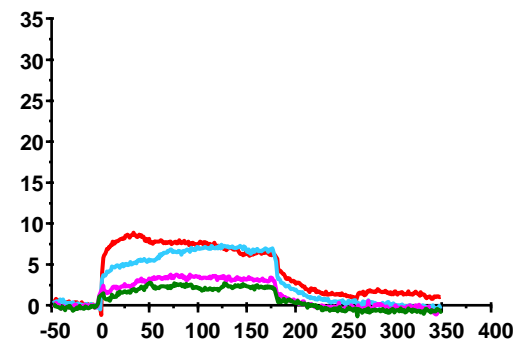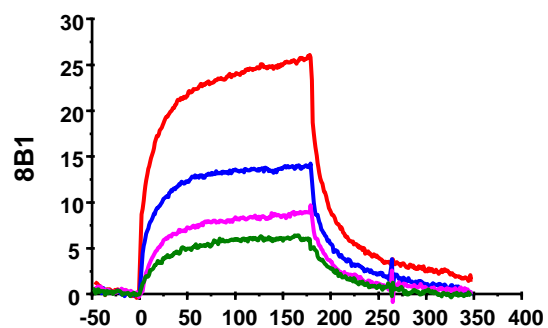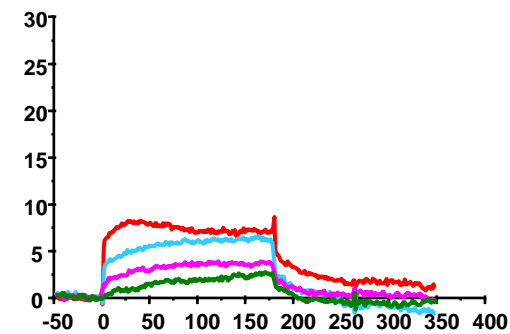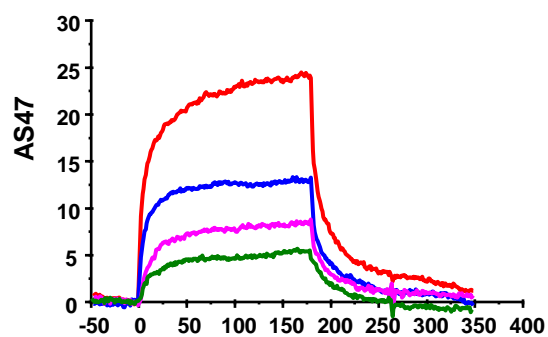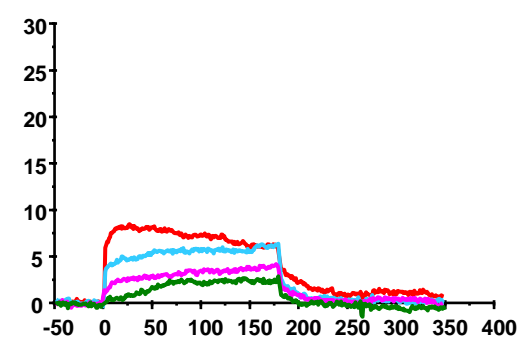

Time (s)

Monomer ( $\mu\text{g/mL}$ )

- 25
- 50
- 100
- 150
- 200

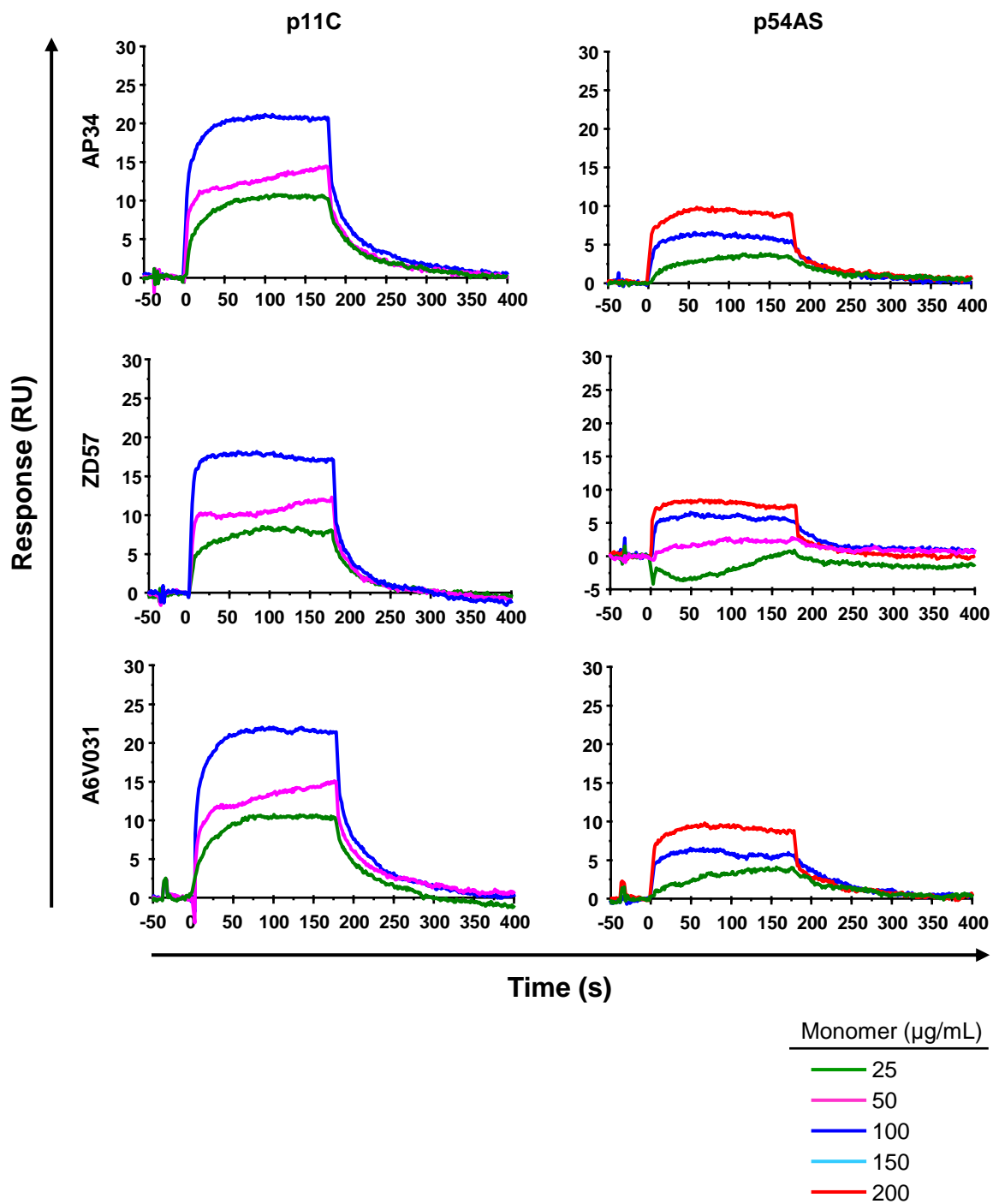

Supplement: Figure S9 — Titrations of p11C and p54E660 peptide:Mamu-A*01 monomers for calculation of binding kinetics and affinity. Shown are sensograms indicating the binding of p11C (left) and p54E660 (right) pMHC monomers to DRMs purified from total CD8+ T cells sorted from seven SIVsmE660-infected monkeys. p11C monomers were run at 25 (green), 50 (pink), 100 (blue), and 200 (red) μg/mL. The ARB0 plot for p11C shows a 150 μg/mL (light blue) run in place of the 100 μg/mL. The AP34, ZD57, and A6V031 plots for p11C do not show the 200 μg/mL run. p54E660 monomers were run at 25, 50, 150, and 200 μg/mL for AP54, ARB0, 8B1, and AS47 and at 25, 100, and 200 μg/mL for AP34, ZD57, and A6V031. The ZD57 plot includes an additional 50 μg/mL run. A Langmuir curve was fit to each binding curve at each concentration and was used to calculate binding kinetics. Readings have been normalized by subtracting the binding of the control monomer TL8 run at the same concentrations as the experimental monomers. (PDF) [file ppat.1004069.s009.pdf]
