# Supplementary material for: TCR Affinity Associated with Functional Differences between Dominant and Subdominant SIV Epitope-Specific CD8+ T Cells in Mamu-A*01 + Rhesus Monkeys
Source: PLoS Pathog. 2014 Apr 17;10(4):e1004069. doi: 10.1371/journal.ppat.1004069 (PMC3990730; doi:10.1371/journal.ppat.1004069)
Supplement: Figure S2 — Individual values of genes differentially expressed between dominant p11C- and subdominant p54AS-specific CD8+ T cells. Shown are the individual normalized expression, measured in fluorescence units, for each A) maturation, B) cytotoxicity, and C) proliferation and apoptosis gene that was determined to be differentially expressed between p11C- and p54AS-specific cells. Plots include values for which its matching pair is missing and therefore were not used in determination of differential expression. Fold change values are indicated in upper left corner. An asterisk under fold change values is present if that gene met the criteria for differential expression on that timepoint. For those genes with more than one probe on the BeadChip, the numerical probe IDs are included in the gene name. Red, p11C. Blue, p54AS. (PDF) [file ppat.1004069.s002.pdf]

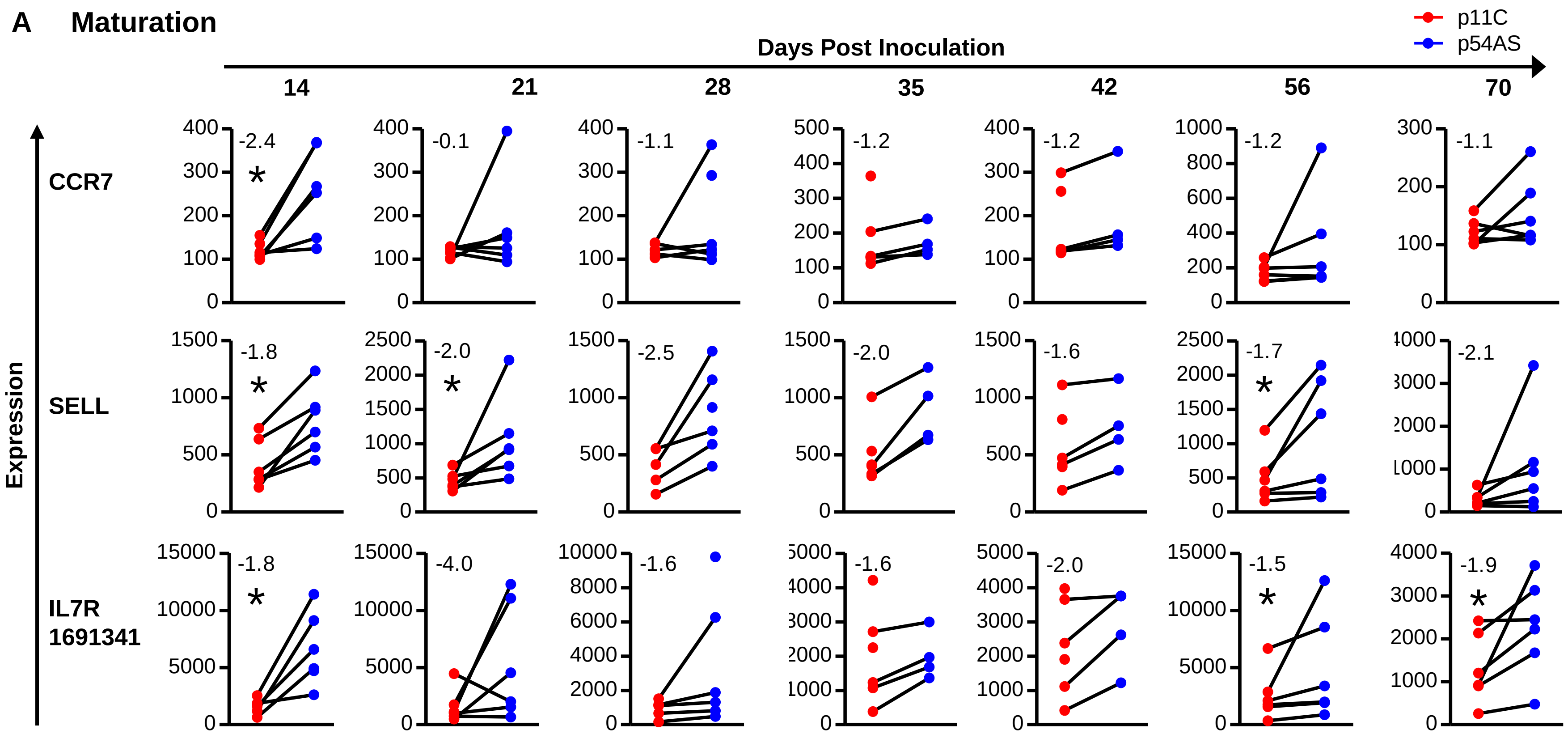

A Maturation
p11C
p54AS
Days Post Inoculation
14
21
28
35
42
56
70
Expression
CCR7
SELL
IL7R 1691341
-2.4
-0.1
-1.1
-1.2
-1.2
-1.2
-1.1
-1.8
-2.0
-2.5
-2.0
-1.6
-1.7
-2.1
-1.8
-4.0
-1.6
-1.6
-2.0
-1.5
-1.9

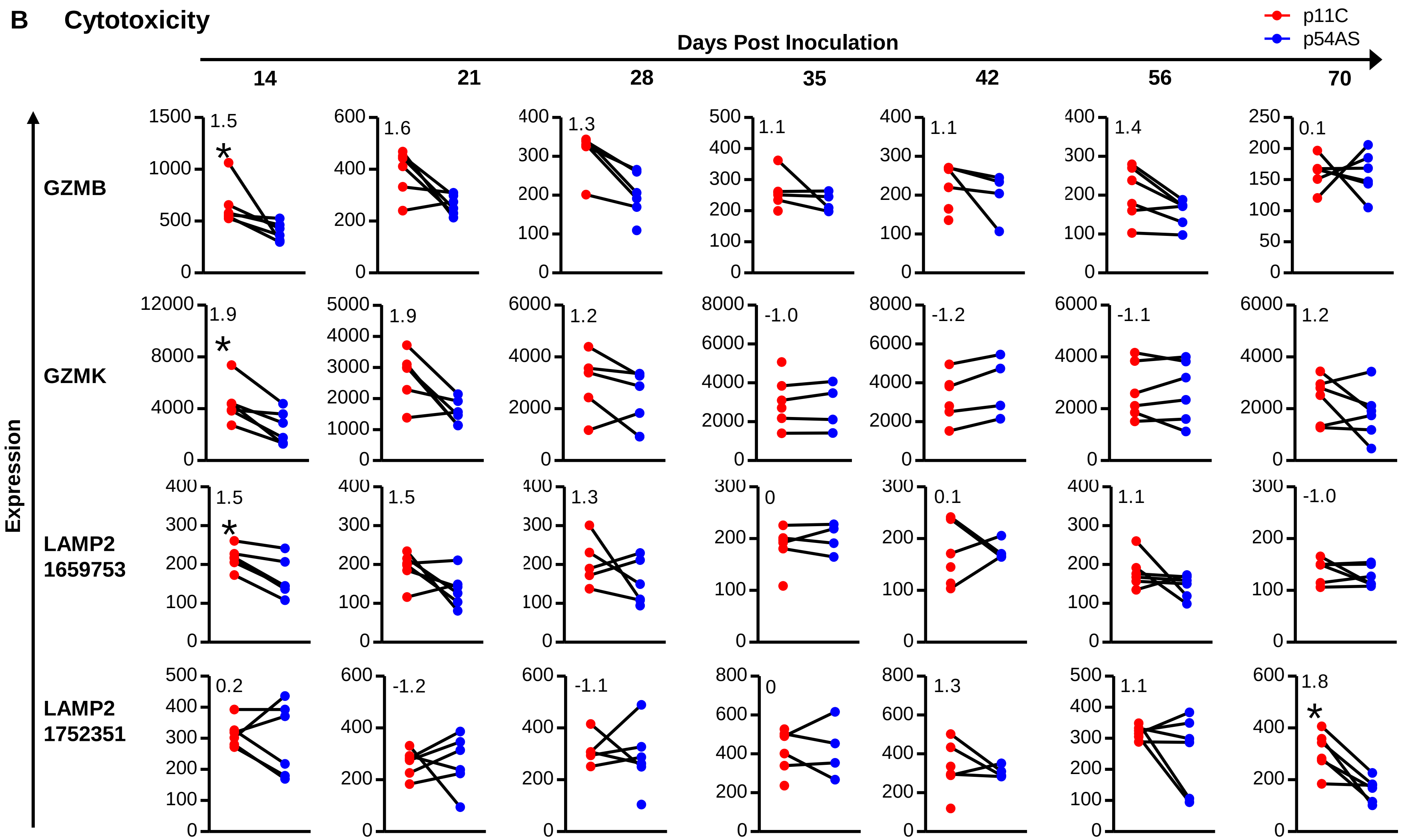
B
Cytotoxicity
p11C
p54AS
Days Post Inoculation
14
21
28
35
42
56
70
Expression
GZMB
1.5
1.6
1.3
1.1
1.1
1.4
0.1
GZMK
1.9
1.9
1.2
-1.0
-1.2
-1.1
1.2
LAMP2 1659753
1.5
1.5
1.3
0
0.1
1.1
-1.0
LAMP2 1752351
0.2
-1.2
-1.1
0
1.3
1.1
1.8
*

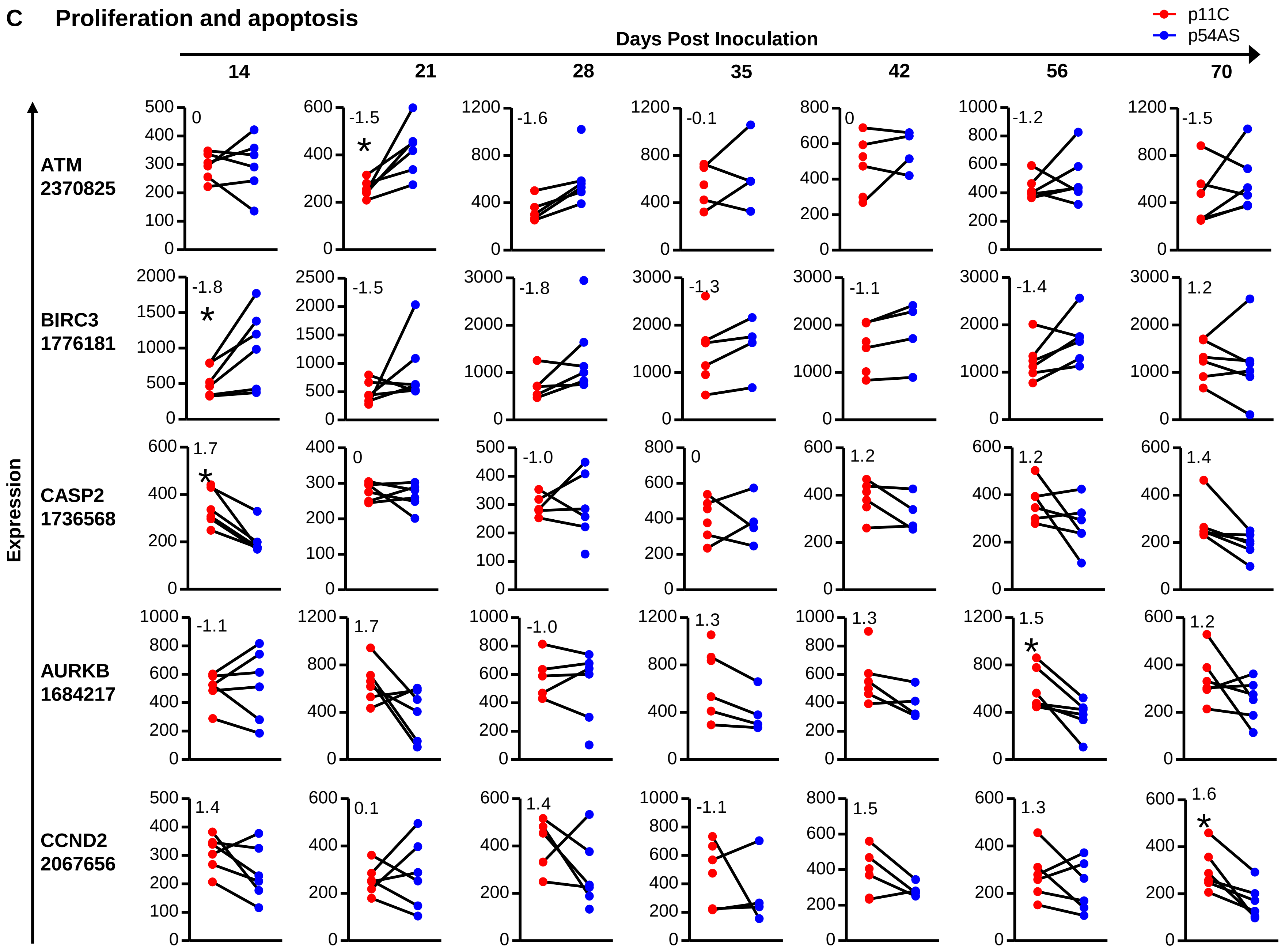
C
Proliferation and apoptosis
p11C
p54AS
Days Post Inoculation
14
21
28
35
42
56
70
Expression
ATM
2370825
BIRC3
1776181
CASP2
1736568
AURKB
1684217
CCND2
2067656
0
-1.5
-1.6
-0.1
0
-1.2
-1.5
-1.8
-1.5
-1.8
-1.3
-1.1
-1.4
1.2
1.7
0
-1.0
0
1.2
1.2
1.4
-1.1
1.7
-1.0
1.3
1.3
1.5
1.2
1.4
0.1
1.4
-1.1
1.5
1.3
1.6
*
